# A positive association between anxiety disorders and cannabis use or cannabis use disorders in the general population- a meta-analysis of 31 studies

**DOI:** 10.1186/1471-244X-14-136

**Published:** 2014-05-10

**Authors:** Karina Karolina Kedzior, Lisa Tabata Laeber

**Affiliations:** 1 School of Humanities and Social Sciences, Jacobs University Bremen, Campus Ring 1, 28759 Bremen, Germany

**Keywords:** Anxiety disorders, Cannabis use, Cannabis use disorders (dependence, abuse), Systematic review, Meta-analysis

## Abstract

**Background:**

The aim of the current study was to investigate the association between anxiety and cannabis use/cannabis use disorders in the general population.

**Methods:**

A total of *N* = 267 studies were identified from a systematic literature search (any time- March 2013) of Medline and PsycInfo databases, and a hand search. The results of 31 studies (with prospective cohort or cross-sectional designs using non-institutionalised cases) were analysed using a random-effects meta-analysis with the inverse variance weights. Lifetime or past 12-month cannabis use, anxiety symptoms, and cannabis use disorders (CUD; dependence and/or abuse/harmful use) were classified according to DSM/ICD criteria or scores on standardised scales.

**Results:**

There was a small positive association between anxiety and either cannabis use (*OR* = 1.24, *95% CI*: 1.06-1.45, *p* = .006; *N* = 15 studies) or CUD (*OR* = 1.68, *95% CI*: 1.23-2.31, *p* = .001; *N* = 13 studies), and between comorbid anxiety + depression and cannabis use (*OR* = 1.68, *95% CI*: 1.17-2.40, *p* = .004; *N* = 5 studies). The positive associations between anxiety and cannabis use (or CUD) were present in subgroups of studies with *ORs* adjusted for possible confounders (substance use, psychiatric illness, demographics) and in studies with clinical diagnoses of anxiety. Cannabis use at baseline was significantly associated with anxiety at follow-up in *N* = 5 studies adjusted for confounders (*OR* = 1.28, *95% CI*: 1.06-1.54, *p* = .01). The opposite relationship was investigated in only one study. There was little evidence for publication bias.

**Conclusion:**

Anxiety is positively associated with cannabis use or CUD in cohorts drawn from some 112,000 non-institutionalised members of the general population of 10 countries.

## Background

The prevalence of affective and anxiety disorders is high, particularly in the western world. According to the annual Health Report 2012 of the second largest national health insurer in Germany (Techniker Krankenkasse), the absence from work due to a diagnosed psychiatric disorder had increased by about 70% from 2000–2012 among clients insured with the company [1]. Next to depression, the most frequent of these diagnoses were different subtypes of anxiety disorders. Although such an extreme increase in prevalence of psychiatric illness was not confirmed based on the national survey data in Germany and other European Union (EU) countries over the same period of time [2], it is clear that psychiatric illnesses are common worldwide and substantially contribute to an overall disease burden. According to nationally-representative samples of adults, particularly the anxiety disorders occur frequently with a lifetime prevalence of <10% in China, Israel, Nigeria, and Japan, between 10-20% in the EU, Ukraine, Mexico, South Africa, and Lebanon, and >20% in France, New Zealand, Columbia, and the highest prevalence of 31% in the USA [2,3]. Not surprisingly, compared to older adults (65+ years of age), the high rates of anxiety disorders are observed especially among the younger adults (18–34 years of age) during the challenging phase of life associated with establishment of professional career paths and long-term relationships/family [3]. Similarly to adults, the 12-month estimates show that anxiety disorders are also the most commonly occurring disorders in 13–17 year old adolescents in the USA [4]. Furthermore, the Australian data (from The National Survey of Mental Health and Wellbeing) suggest that more females (32%) than males (20%) experience anxiety disorders (especially the post-traumatic stress disorder, PTSD) in their lifetime [5].

After alcohol and nicotine, cannabis is the most widely consumed illicit substance [6,7] with an estimated 13.1 million dependent users worldwide in 2010 [8]. Interestingly, the highest estimated cumulative incidence of cannabis use (42%) was observed in the two countries with the highest rates of anxiety disorders- the USA and New Zealand [6]. These findings suggest that anxiety and cannabis use might be related either directly or indirectly via common factors. However, the empirical evidence so far suggests that, among psychiatric disorders, cannabis use is most consistently associated only with psychosis [9]. According to meta-analyses, such an association is particularly strong in frequent users compared to ever users [10], early cannabis use increases the odds of later psychosis [11], cannabis use (independent of gender or heaviness of use) is related to an earlier onset of psychosis by about 3 years [12], and cannabis use disorders are observed in particularly younger, male, first-episode patients with schizophrenia [13]. In contrast, heavy or problematic cannabis use was only moderately related to depression outcomes [10,14,15] and anxiety [16]. However, such associations were often eliminated after controlling for confounding factors, such as other substance use and/or other psychiatric comorbidity [16-18]. This result is not surprising because cannabis use is strongly associated with the use of other substances, especially nicotine [19], and nicotine use and dependence are related to some anxiety disorders, such as panic disorder and generalised anxiety disorder [20]. Similarly, clinical comorbidities frequently exist among different subtypes of anxiety disorders and depression [21] or alcohol use disorders [22]. Regardless of these possible confounders, a systematic, quantitative assessment of comorbidity between anxiety and cannabis use is needed because both conditions commonly occur in the general population, particularly during the most productive yet stressful life-phases (adolescent-middle age). If the two conditions coexist after controlling for confounders then this result might have some implications for clinical treatment and policy making. Specifically, the methods of detection and treatment of both conditions concurrently might need to be reassessed to provide effective patient care [23]. Such treatment could reduce any misdiagnoses because the acute effects of cannabis use resemble symptoms of anxiety disorders, such as panic attacks [16]. However, those seeking treatment for symptoms of anxiety may not disclose their cannabis use due to the illegal nature of this activity [14]. Furthermore, the link to anxiety could also be used as supplementary evidence for development of a rational cannabis policy that currently differs among countries worldwide [24].

To the best of our knowledge, only one systematic review so far attempted to quantify the association between cannabis use and anxiety disorders [10]. The authors listed the effect sizes based on *N* = 7 studies using nationally-representative cohorts and focusing on the association between ‘cannabis exposure’ and ‘anxiety outcomes’ (Figure six of the article). However, due to a high heterogeneity among these seven studies, their effect sizes were not combined quantitatively in a meta-analysis. The studies were not comparable because they utilised different classifications of anxiety disorders (either anxiety alone or comorbid anxiety and depression) and cannabis use (either use vs. no use or use with vs. without cannabis use disorders, such as dependence or abuse/harmful use). Therefore, it is not surprising that the effect sizes in the seven studies were highly inconsistent and did not suggest any general trend towards presence or absence of a relationship between anxiety and cannabis use [10].

The aim of the current study was to quantitatively assess the relationship between anxiety and cannabis use by means of a systematic literature review followed up by a quantitative meta-analysis. In contrast to Moore and colleagues [10], our aim was to include not only the large longitudinal studies but also smaller cross-sectional studies. The reasons for including such studies were to improve the statistical power of meta-analysis and to perform the analyses on subgroups of more homogenous studies. Specifically, our aim was to conduct the statistical meta-analysis on subgroups of studies based on similar classification of anxiety disorders (anxiety alone vs. comorbid anxiety and depression) and cannabis use (use vs. no use or use with vs. without cannabis use disorders). Finally, it was of interest to find out if an adequate volume of studies exists to test the direction of the main relationship. Specifically, our aim was to compute the odds for cannabis use at follow-up in cohorts with baseline anxiety and the odds for anxiety at follow-up in cohorts with baseline cannabis use.

It was hypothesised that a positive relationship exists between anxiety disorders and cannabis use (particularly with cannabis use disorders) based on the positive relationships between particularly heavy cannabis use and other psychiatric diagnoses (such as psychosis and depression). We expected that some evidence for the direction of such a relationship could emerge from the analysis of prospective studies. Specifically, we could establish which baseline condition (anxiety or cannabis use) would be related to higher odds of the other condition at follow-up. Furthermore, it was expected that the association between anxiety and cannabis use (or cannabis use disorders) would be lower (or even absent) in studies with effect sizes adjusted for confounding factors, such as other substance use, other psychiatric comorbidity, and demographics (age, gender), compared to studies with unadjusted effect sizes. Finally, it cannot be ruled out that the use of more advanced quantitative models to establish prevalence of comorbid conditions had some influence on the effect sizes reported in primary studies over time. Thus, we expected that there would be a negative relationship between effect sizes and the date of publication of studies (regardless of when the data were collected). Specifically, we hypothesised that the effect sizes would be adjusted for more factors using more complex statistical models (and thus be lower) in the newer than in the older studies.

## Methods

### Systematic literature search

A systematic literature search was conducted in March 2013, according to the steps depicted in Table 1.

**Table 1:** Details of the systematic literature search (all searches conducted in English with no language restrictions)

**Search**	**Search terms**	**Databases and timeframes**
Search 1 *N* = 131	Subject OR Title (cannabis or marijuana or marihuana) AND Subject OR Title (“affective disorder” or “anxiety disorder” or anxiety) NOT (mouse or mice or rat or rats)	PsycInfo (1806-March 2013)
Search 2 *N* = 168	Subject OR Title (cannabis or marijuana or marihuana) AND Subject OR Title (“affective disorder” or “anxiety disorder” or anxiety) AND Keyword (misus* or abus* or depend* or "harmful use" or "harmful usage") NOT (mouse or mice or rat or rats)	Medline (1950-March 2013)
***N*** = 256	Total *N* from both searches excluding duplicates	

Note: Additional *N* = 11 studies were obtained from other meta-analyses being conducted by the authors, hand-searches of bibliographies of retrieved sources, and from the bibliography of Moore *et al.*[10].

*N* = number of sources identified during each search.

A total of *N* = 267 sources identified during the search (Table 1) were assessed and *N* = 218 studies were excluded (the reasons for exclusion are summarised in the Additional file 1: Table S1).

### Study selection

**Figure 1:**
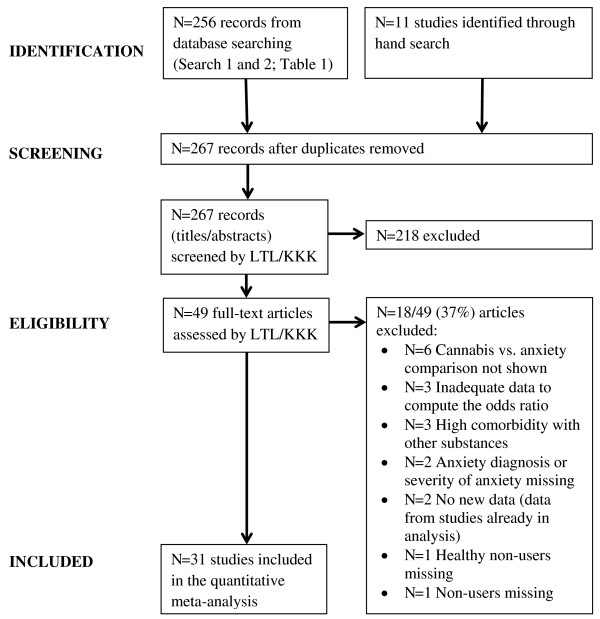
Selection of studies and exclusion criteria.

The summary of the study selection procedure and exclusion criteria are shown in the PRISMA flowchart [25], Figure 1.

Of *N* = 267 sources, *N* = 49 studies were retrieved and assessed in full-length. All these studies and, if applicable, the reasons for their exclusion are listed in the Additional file 1: Table S2. A total of *N* = 31 studies were selected for inclusion in the final analysis. Studies were included if they reported:

(1) Data based on samples from a non-institutionalised, general population;

(2) Anxiety diagnoses (with or without comorbid depression), based on DSM/ICD diagnostic criteria, and/or anxiety severity score, based on standardised scales, in cannabis users/non-users or cannabis users with/without cannabis use disorder (CUD). CUD was defined as dependence and/or abuse/harmful use according to DSM/ICD criteria;

(3) Cannabis use/no use (or cannabis use with/without CUD) in cases with anxiety/no anxiety;

(4) Odds ratios, *ORs,* and their 95% confidence intervals, *95% CI* (or sufficient information to compute these values), for

● anxiety/no anxiety in cannabis users/non-users or cannabis users with/without CUD or

● cannabis use/no use or cannabis use with/without CUD in cases with anxiety/no anxiety;

(5) Data sufficient to compute other effect sizes, that were later converted into *OR*, such as the standardised mean difference (Cohen’s *d*), based on means, *SD*, and *N* of severity of anxiety scores in cannabis users/non-users (or cannabis users with/without CUD).

Studies were excluded if they:

(1) Did not report data from healthy non-users;

(2) Reported data from samples seeking treatment for CUD and/or with a high comorbidity of other-substance use and/or other concurrent psychiatric disorders than anxiety and depression;

(3) Reported inadequate data to compute any effect size (unless the authors provided additional data).

### Data extraction

The data from *N* = 31 studies were extracted/double-checked for consistency by both authors.

The characteristics of all *N* = 31 studies and the effect size data from these studies are listed in Tables 2 and 3. To maintain a high consistency of results the following data extraction rules were used:

(1) Only the most conservative estimates of *ORs* were extracted (i.e. the *ORs* adjusted for as many confounders as possible). If data based on the same cases were presented in more than one study then the study with more conservative *ORs* was selected for the final analysis;

(2) Data based on the longest possible time-spans (lifetime) were extracted;

(3) Data based on the heaviest use and/or cannabis dependence were extracted;

(4) Consistent estimates were extracted to combine *ORs* from different studies using data from the same cases (e.g. lifetime PTSD and lifetime panic disorder from two NCS-R studies, see Table 3);

(5) As many types of anxiety disorders as possible were included if *ORs* for individual anxiety disorders were reported separately for different groups of cases.

**Table 2:** **Characteristics of *N*** **= 31 studies included in the current meta-analysis**

**Study; Country**	Design*	**Total** ***N *** **(*N *** in this study**)	**Sample type**	**Sampling method**	**Anxiety assessment; diagnostic system**	**Anxiety diagnosis (timeframe)**	**Anxiety prevalence**	**Cannabis assessment; diagnostic system**	**Cannabis use; CUD (timeframe)**	**Cannabis use/CUD prevalence**
Agosti *et al.,* 2002; NCS, USA [26]	Cross-sectional	8098	General population	Probability	CIDI DSM-III-R	AD (current)	–	CIDI DSM-III-R	Use (past month); CD (lifetime)	–
Beard *et al.,* 2006; NoRMHS, Australia [27]	Longitudinal	9191 (1013)	General population	Random	CIDI ICD-10	PD, SP, OCD (at T1)	15% (at T1)	CIDI ICD-10	CUD (at T0)	CUD 3% (at T0)
Brook *et al.,* 1998; Upstate NY, USA [28]	Longitudinal	975 (T3 = 745 T4 = 698)	Adolescents- adults	Random	DISC DSM-III-R	SA, OAD, SAD (at T4)	–	DISC DSM-III-R	Never- ≥ weekly (at T3)	Use (at T3): 56% never 9% ≥ weekly
Brook *et al.,* 2001; Colombia [29]	Longitudinal	2226	Adolescents	Random	HSC	AD (at T1, T2)	–	Interview	Non-regular- regular (≥monthly; lifetime)	–
Buckner *et al.,* 2008; Oregon, USA [30]	Longitudinal	T1 = 1709 T4 = 816	High school students	Random	K-SADS DSM-III-R	SAD (at T1)	2% (at T1)	LIFE, SCID-I/NP DSM-IV	CD (at T4)	CD 6% (at T4)
Buckner & Schmidt, 2008; USA [31]	Cross-sectional	337 (214)	Undergraduate students	Random	SIAS	Equivalent to SAD	19% (scores in clinical range)	Questionnaire	Never- frequent (≥weekly; lifetime)	31% never 32% frequent
Buckner *et al.,* 2012; USA [32]	Cross-sectional	343 (200)	Adult tobacco smokers	Random	SIAS	Equivalent to SAD	–	Questionnaire	Never- current (daily; past month)	19% never 39% current use; 13% current daily
Cascone *et al.,* 2011; Switzerland [33]	Cross-sectional	110	Adolescents in schools/psycho-educational unit	Convenience	STAI-Y B	Trait anxiety	–	ADAD DSM-IV-TR	Use (past month) CD (past 12 months)	Past month: No CD: 94% none CD: 66% daily
Chabrol *et al.,* 2005; France [34]	Cross-sectional	212	High school and college students	Random	STAI A	State anxiety	–	Questionnaire DSM-IV	Past 6 months: None- > daily; CD	46% none 23% > daily
Chabrol *et al.,* 2008; France [35]	Cross-sectional	248	High school students	Random	STAI A	State anxiety	–	Interview	None- use ≥1× (past 6 months)	76% none 24% users
Cheung *et al.*, 2010; CAMH, Canada [36]	Cross-sectional cycles of 2001–2006 survey	14531 (13478)	General population	Probability	GHQ12 ≥4/12 symptoms	AMD (past 12 months)	9% (past 12 months)	Interview	None- daily (past 12 months)	–
Cougle *et al.,* 2011; NCS-R, USA [37]	Cross-sectional	5672	General population	Stratified probability	CIDI DSM-IV	PTSD (lifetime)	7% (lifetime)	CIDI	Never- use ≥1× (lifetime)	42% users (lifetime)
Crum *et al.,* 1993; ECA, USA [38]	Longitudinal	18572 (577)	General population	Probability	DIS DSM-III	OCD (past 12 months)	1% (past 12 months)	DIS	No use- use ≥6× (past 12 months)	84% no drug use 9% cannabis
Degenhardt *et al.*, 2001; NSMHWB, Australia [39]	Cross-sectional	10641	General population	Stratified random	CIDI DSM-IV	SAD, AP, PD, GAD, OCD, PTSD (past 12 months)	6% (past 12 months)	CIDI DSM-IV	Past 12 months: No use- use ≥5×; CD	Past 12 months: 5% users 2% CD
Degenhardt *et al.,* 2010; VAHCS, Australia [40]	Longitudinal	1943 (1520, wave 1–8)	High school students	Stratified random	GHQ12 > 2 symptoms (at 24)	AMD (at 24)	21% (at 24)	Interview	None- weekly+ (past 6 months at 15–17)	34% users (at 15–17)
Degenhardt *et al.,* 2013; VAHCS, Australia [41]	Longitudinal	1943 (1756, wave 1–9)	High school students	Stratified random	CIDI ICD-10	GAD, SAD, PD, AP (past 12 months at 29)	11% (at 29)	CIDI ICD-10	None/<weekly- weekly + (past 6–12 months at 15–29); CD (past 12 months at 29)	Lifetime: 67% none/ <weekly 2% weekly+ 4% CD (at 29)
Fergusson *et al.,* 1996; CHDS, New Zealand [42]	Longitudinal	1265 (927)	Adolescents (16 years)	Stratified	DISC/DIS DSM-III-R	GAD, OAD, SA (at 15–16)	9% (at 15–16)	Interview	None- use (past 12 months at 15–16)	20% users (at 15–16)
Hayatbakhsh *et al.*, 2007; MUSP, Australia [43]	Longitudinal	7223 (3157)	Adult children of mothers in study	Convenience	YASR (resembles DSM-III-R)	AMD (at 21)	–	Interview	Never used drugs- frequent ≤ daily (past month)	12% frequent
Lamers *et al.*, 2006; USA [44]	Cross-sectional	41 (30)	General population	Convenience	BAI	BAI scores	–	Questionnaire urine screen	None (past 12 months)- use ≥10× (lifetime)	50% non-users 50% users
Low *et al.,* 2008; USA [45]	Cross-sectional	632	Adolescents in primary care	Convenience	PRIME-MD DSM-IV	PD, GAD, AD (past 1–6 months)	7% (past 1–6 months)	PRIME-MD DSM-IV	CA (past 6 months)	6% CA
Martins & Gorelick, 2011; NESARC, USA [46]	Cross-sectional	43093	General population	Stratified random	AUDADIS DSM-IV	PD, AP, SP, GAD (lifetime)	17% (lifetime)	AUDADIS DSM-IV	CA + CD (lifetime)	–
McGee *et al.*, 2000; DMHDS, New Zealand [47]	Longitudinal	1037 (891)	Adolescents (at 15)	Convenience	DISC DSM-III	Internalising disorders (AMD; at 15)	12% (at 15)	Interview	None- use ≥1 (past 12 months)	14% users (at 15)
NPMS, UK; appendix, Moore *et al.*, 2007 [10]	Longitudinal	8580 (1578)	Adults (at 16–74)	Random	CIS-R ≥ 12	AMD	11% CIS-R ≥12	Interview	Use: no/yes (lifetime); CD (past 12 months): no/yes	16% users 2% CD
Patton *et al.,* 2002; VAHCS, Australia [48]	Longitudinal	1943 (1601, wave 1–7)	High school students	Stratified random	CIS-R ≥ 12 at 21	AMD (at 21)	16% (at 21)	Interview	None- < weekly (past 6 months at 15–17)	59% users (lifetime)
Roberts *et al.,* 2007; TH2K, USA [49]	Cross-sectional	4175	Adolescents	Probability	DISC DSM-IV	AP, GAD, PD, SAD, PTSD (past 12 months)	7% (past 12 months)	DISC DSM-IV	CUD (past 12 months)	3% CUD (past 12 months)
Swift *et al.,* 2008; VAHCS, Australia [50]	Longitudinal	1943 (1520, wave 1–8)	High school students	Stratified random	CIS-R > 11	AMD (at 15–17)	–	Interview, CIDI DSM-IV	Past 12 months at 24: None- weekly+; CD	28% weekly+ at 24 who used at 15-17
van der Pol *et al.,* 2013; CanDep + NEMESIS-2, Netherlands [51]	Cross-sectional	1324: D+: 252 N2: 1072	General population/ ‘coffee shop’ users (18–30)	Stratified random; convenience/chain-referral	CIDI DSM-IV	SAD, PD, GAD, AP (past 12 months)	8% (past 12 months)	CIDI DSM-IV	No CD (group N2; none or <3×/week use)- CD (D+; use ≥3×/week) (past 12 months)	16% CD (past 12 months)
Van Laar *et al.,* 2007; NEMESIS, Netherlands [52]	Longitudinal	T0: 7076 T2: 4848	General population	Probability	CIDI DSM-III-R	PD, AP, SAD, SP, GAD, OCD (3-year incidence, T0-T2)	6% (at T0-T2)	CIDI DSM-III-R	No use- use >5× (lifetime at T0)	–
Wittchen *et al.*, 2007; EDSP, Germany [53]	Longitudinal	T0: 1395 T3: 1019 (1310)	General population	Random	CIDI DSM-IV	PD, GAD, AP, SAD, SP, SA, OCD, PTSD (at T0)	23% (at T0)	CIDI DSM-IV	Use: no/yes (lifetime); CUD (lifetime)	Lifetime: 54% use 13% CUD
Zvolensky *et al.,* 2006; CSHS, USA [54]	Cross-sectional	4745	General population	Stratified random	DIS DSM-IV-TR	PA (lifetime)	6% (lifetime)	DIS DSM-IV-TR	Lifetime: No use- use ≥5×; CD	Lifetime: 25% users 1% CD
Zvolensky *et al.,* 2010; NCS-R, USA [55]	Cross-sectional	5672	General population	Stratified probability	CIDI DSM-IV	PD (lifetime)	6% (lifetime)	CIDI	None- use ≥1× (lifetime)	42% users (lifetime)

Notes: All studies included males and females of any race. *Abbreviations:* AD = anxiety disorder; ADAD = Adolescent Drug Abuse Diagnosis (based on Addiction Severity Index); AMD = anxiety + depression; AP = agoraphobia; AUDADIS = Alcohol Use Disorders and Associated Disabilities Interview Schedule; BAI = Beck Anxiety Inventory; CA = cannabis abuse; CAMH = Centre for Addiction and Mental Health Monitor survey, Canada; CanDep = the Dutch Cannabis Dependence Study, Netherlands; CD = cannabis dependence; CHDS = Christchurch Health and Development Study, New Zealand; CIDI = Composite International Diagnostic Interview; CIS-R = Clinical Interview Schedule- Revised; CSHS = Colorado Social Health Survey, USA; CUD = cannabis use disorder (abuse/harmful use and/or dependence); D + = frequent cannabis users with dependence in CanDep study; DIS = Diagnostic Interview Schedule; DISC = Diagnostic Interview Schedule for Children; DMHDS = Dunedin Multidisciplinary Health and Development Study, Dunedin, New Zealand; ECA = Epidemiological Catchment Area program, USA; EDSP = Early Developmental Stages of Psychopathology study, Germany; GAD = generalized anxiety disorder; GHQ-12 = General Health Questionnaire (12 items); HSC = Hopkins Symptom Checklist; K-SADS = Schedule for Affective Disorders and Schizophrenia for School-Age Children; LIFE = Longitudinal Interval Follow-up Evaluation; MUSP = Mater University Study of Pregnancy, Brisbane, Australia; NCS = National Comorbidity Survey, USA; NCS-R = National Comorbidity Survey- Replication, USA; NEMESIS/NEMESIS-2 = Netherlands Mental Health Survey and Incidence Study (study 1: 1996–1999 and study 2: 2007–2009), N2 = NEMESIS-2 cases; Netherlands; NESARC = National Epidemiological Survey on Alcohol and Related Conditions, USA; NoRMHS = the Northern Rivers Mental Health Study, New South Wales, Australia; NPMS = the British National Psychiatric Morbidity Survey, UK; NSMHWB = National Survey of Mental Health and Well-Being, all states, Australia; OAD = overanxious disorder; OCD = obsessive compulsive disorder; PA = panic attacks; PD = panic disorder; PRIME-MD = Primary Care Evaluation of Mental Disorders; PTSD = post-traumatic stress disorder; SA = separation anxiety; SAD = social anxiety disorder/social phobia; SCID-I/NP = Structured Clinical Interview for DSM-IV, non-patient version; SIAS = Social Interaction Anxiety Scale; SP = specific phobias; STAI = State-Trait Anxiety Inventory; STAI-Y = STAI for Youth; STAI-Y A = STAI state anxiety subscale; STAI-Y B = STAI trait anxiety subscale; T = specific wave of data collection in longitudinal studies; TH2K = Teen Health 2000 Study, Houston, USA; VAHCS = Victorian Adolescent Health Cohort Study, Victoria, Australia; YASR = Young Adult Self-Report.

*Cross-sectional was chosen if the results were obtained from one data set (even if the study was longitudinal), longitudinal refers to studies that show data at different time points (waves).

**Most studies did not specify the total *N* used to compute the *ORs* used in the current study.

**Table 3:** **Odds ratios (*OR *) for anxiety (or anxiety + depression) vs. no disorder in cannabis users vs. non-users (or in cannabis users with CUD vs. no CUD) in *N*** **= 31 studies**

**Study/Name (Part 1: anxiety diagnoses)**	**Cannabis use vs. no use**		**CUD vs. no CUD (or no use)**	***OR*** **(*95% CI *) Anxiety/no anxiety in user/non-user**	***OR*** **(*95% CI *) Anxiety/no anxiety in CUD/no CUD**	**Data location in study**	***OR*** **adjusted for**
Agosti *et al.*, 2002; NCS, USA [26]			Current AD in lifetime CD who used within past month vs. no CD		2.6 (1.5-4.5)	Text p. 646	No information
Beard *et al.*, 2006; NoRMHS, Australia [27]^1^			T0 CUD (vs. no CUD) to T1 AD		.78 (.18-3.30)	Table three	Unadjusted (comorbidity with other diagnoses possible)
Brook *et al.*, 1998; Upstate NY, USA [28]	T4 AD to T3 use vs. no use			1.16 (1.00-1.35)		Table two	Demographics, prior AD
Brook *et al.*, 2001; Columbia [29]^2^	T1 AD to T2 regular vs. non-regular use			.94 (.86-1.03)		Table one	Demographics, cannabis use at T1
	T1 regular vs. non-regular use to T2 AD			1.48 (1.09-2.01)		Table two	Demographics, AD at T1
	Combined: AD or regular use: T1 vs. T2			1.18 (.94-1.48)			
							
Buckner *et al.,* 2008; Oregon, USA [30]			T1 SAD to T4 CD vs. no CD		4.88 (1.43-16.64)	Text p. 235	Gender, T1 anxiety, conduct, mood, alcohol use disorders, T1 CUD excluded
Cascone *et al.,* 2011; Switzerland [33]			Past 12 months CD (vs. no CD) predicted with STAI-Y B		1.02 (.97-1.08)	Table five	Withdrawal coping, social problems, recruitment context
Cougle *et al.,* 2011; NCS-R, USA [37]^3^	Lifetime: PTSD to use vs. no use			2.45 (1.70-3.52)		Table one	Demographics, lifetime alcohol/nicotine dependence/abuse
Crum *et al.,* 1993; ECA, USA [38]^4^	Past 12 months: OCD to use vs. no use			1.54 (.78-3.04)		Table one	Excluded past or baseline OCD cases
Degenhardt *et al.,* 2001; NSMHWB, Australia [39]	Past 12 months: AD to use vs. no use		Past 12 months: AD to CD vs. no use	.88 (.60-1.29)	1.40 (.84-2.37)	Table four	Demographics, other substance use, neuroticism
Degenhardt *et al.,* 2013; VAHCS, Australia [41]	AD at 29 to ≥ weekly vs. no use at 15-29		AD at 29 to CD at 29 vs. no CD	3.2 (1.1-9.2)	2.2 (1.1-4.4)	Table one, three	Demographics, alcohol/substance use at 29, adolescent anxiety/depression
Fergusson *et al.,* 1996; CHDS, New Zealand [42]	AD at 16 to use vs. no use at 15			1.2 (.5-2.8)		Table three	Demographics, substance use and dependence, anxiety/depression, other mental health problems at 15
Low *et al.,* 2008; USA [45]			Past 6 months: CA (vs. no CA) to AD		1.4 (.4-4.7)	Table four	Demographics, sampling site, depression
Martins & Gorelick, 2011; NESARC, USA [46]			Lifetime: CUD (vs. no CUD) to AD		3.2 (2.0-5.2)	Table four	Demographics
Roberts *et al.,* 2007; TH2K, USA [49]			Past 12 months: AD to CUD vs. no CUD		.9 (.4-2.1)	Table six	Concurrent (past 12 months) mood, conduct, ADHD disorders; alcohol, other substance abuse/dependence
van der Pol *et al.,* 2013; CanDep + NEMESIS-2, Netherlands [51]			Past 12 months: AD to CD (vs. no/non-frequent use) (D + vs. N2 groups)		1.12 (.48-2.63)	Table two; Authors	Demographics, childhood adversity, tobacco (past month), frequent alcohol, cocaine, ecstasy use (past 12 months)
van Laar *et al.,* 2007; NEMESIS, Netherlands [52]	AD (T0-T2) to T0 use vs. no use			1.18 (.71-1.97)		Table four	Demographics, neuroticism, childhood trauma, lifetime: alcohol, other SUDs, psychotic symptoms, AD
Wittchen *et al.,* 2007; EDSP, Germany [53]	Lifetime use vs. no use to T0 AD		Lifetime CUD vs. no CUD to T0 AD	1.5 (1.1-2.1)	1.7 (1.1-2.5)	Table four	Gender
Zvolensky *et al.,* 2006; CSHS, USA [54]	Lifetime: use vs. no use to PA		Lifetime: CD vs. no CD to PA	.89 (.63-1.30)	2.1 (1.1-4.3)	Text p. 482	Demographics, other substance use
Zvolensky *et al.,* 2010; NCS-R, USA [55]^3^	Lifetime: PD to use vs. no use			1.70 (1.33-2.17)		Table one	Demographics, lifetime alcohol, nicotine, illicit substance abuse/dependence
NCS-R combined^3^	Lifetime: PTSD + PD to use vs. no use			2.04 (1.50-2.78)			
Study/Name (Part 2: anxiety severity scores)*	**Non-user** ***M (SD); N *** **(timeframe)**	**Cannabis User** ***M (SD); N*** **(timeframe)**	**CUD** ***M (SD); N *** **(timeframe)**	***OR*** **(*95% CI *) Anxiety/no anxiety in user/non-user**	***OR*** **(*95% CI *) Anxiety/no anxiety in CUD/no CUD**	**Location in study**	**Scale (study exclusionary criteria)**
Buckner & Schmidt, 2008; USA [31]	23.6 (11.6); 105 (lifetime)	22.2 (13.1); 109 (≥weekly frequent use; lifetime)		.81 (.50-1.33)		Table one	SIAS (none)
Buckner *et al.,* 2012; USA [32]	21.3 (15.5); 66 (lifetime)	21.7 (13.7); 134 (past month)		1.05 (.62-1.80)		Table one; Authors	SIAS (high-risk suicidal behaviour, psychotic)
Chabrol *et al.,* 2005; France [34]^5^	35.3 (11.1); 98 (past 6 months)	37.1 (10.3); 114 (past 6 months)	38.1 (9.8); 44 (past 6 months)	1.36 (.83-2.22)	1.61 (.84-3.07)	Table two Table three	STAI A (none)
Chabrol *et al.,* 2008; France [35]	38.3 (12.4); 189 (past 6 months)	42.9 (13.3); 59 (past 6 months)		1.94 (1.14-3.30)		Table one	STAI A (none)
Lamers *et al.,* 2006; USA [44]	4.0 (4.3); 15 (past 12 months)	2.9 (1.9); 15 (lifetime)		.55 (.15-2.03)		Table three	BAI (alcohol, drug dependence, schizophrenia, depression, antisocial behaviour, psychoactive drug use)
**Study/Name (Part 3: anxiety + depression, AMD)**	**Cannabis use vs. no use**		**CUD vs. no CUD (or no use)**	***OR*** **(*95% CI *) AMD/no AMD in user/non-user**	***OR*** **(*95% CI *) AMD/no AMD in CUD/no CUD**	**Location in study**	***OR*** **adjusted for**
Cheung *et al.*, 2010; CAMH, Canada [36]	Past 12 months: AMD to daily use vs. no use			2.05 (1.18-2.93)		Table two	Demographics, alcohol misuse
Degenhardt *et al.*, 2010; VAHCS, Australia [40]^6^	AMD at 24 (wave 8) to weekly + use vs. no use past 6 months at 15–17 (wave 1–6)			.88 (.55-1.40)		Table two	Demographics, adolescent: AMD, alcohol, nicotine use
Hayatbakhsh *et al.*, 2007; MUSP, Australia [43]	AMD at 21 to frequent (past month) vs. never used drugs (lifetime)			2.1 (1.1-4.0)		Table four	Demographics, no other illicit drugs, maternal and adolescent: AMD, alcohol, nicotine use
McGee *et al.*, 2000; DMHDS, New Zealand [47]^7^	Internalising disorders at 15 to use vs. no use past 12 months at 15			2.45 (1.41-4.25)		Table five	Unadjusted (adjusted *OR* could not be used because *95% CI* were not reported)
NPMS, UK; appendix, Moore *et al.*, 2007 [10]	AMD (CIS-R ≥ 12) to ever use vs. no use		AMD (CIS-R ≥ 12) to CD vs. no CD	.8 (.4-1.6)	.9 (.2-3.6)	p. IV	Excluded if baseline CIS-R ≥ 12, demographics, other drugs, alcohol, nicotine use
Patton *et al.,* 2002; VAHCS, Australia [48]^6^	AMD (CIS-R ≥ 12) at 21 (wave 7) to < weekly use vs. no use past 6 months at 15–17 (wave 1–6)			1.4 (.94-2.0)		Table three	AMD at 15–17, alcohol use, parental demographics
Swift *et al.,* 2008; VAHCS, Australia [50]^6^	Weekly + use vs. no use (past 12 months at 24, wave 8, who used cannabis at 15–17, waves 1–6) to AMD (CIS-R > 11) at 15–17 (at 3/6 waves of wave 1–6)		CD vs. no CD (past 12 months at 24, wave 8, who used cannabis at 15–17, waves 1–6) to AMD (CIS-R> 11) at 15–17 (at 3/6 waves of wave 1–6)	2.0 (1.0-3.8)	1.4 (.71-2.9)	Table four	Demographics, adolescent: maximum level of cannabis use, nicotine and alcohol use, antisocial behaviour
VAHCS combined^6^	AMD at 15–24 to at least < weekly use at 15–17 (vs. no use)			1.35 (.80-2.27)			

Notes: For abbreviations refer to Table 2.

^1^The *OR* was computed based on the following *N* of cases in the ‘Anxiety’ column and ‘Baseline No Diagnosis’ and ‘Cannabis Diagnosis’ rows reported in Table three of the article: *N* = 2 (CUD/anxiety), *N* = 46 (CUD/no anxiety), *N* = 51 (no CUD/anxiety), *N* = 914 (no CUD/no anxiety).

^2^The two *ORs* were combined according to the formulae for combining dependent effect sizes shown in the Additional file 1.

^3^Both studies reported *ORs* based on the same number of cases from the same study (NCS-R). It was assumed that both studies were dependent (same cases might have been used to compute the *ORs* in both studies). Thus, both *ORs* were combined into one common *OR* that was used in all subsequent analyses using the formulae shown in the Additional file 1.

^4^The *OR* was computed based on the following *N* of cases in the ‘Cases’ (OCD) vs. ‘Non-cases’ columns and ‘Use of marijuana only’ and ‘No drug use’ rows reported in Table one of the article: *N* = 12 (use/anxiety), *N* = 42 (use/no anxiety), *N* = 82 (no use/anxiety), *N* = 441 (no use/no anxiety). The risk ratio (*RR*), adjusted for confounders, was also reported in the study (Table two). However, *RR* and *OR* are not equivalent [56] and thus unadjusted *OR* is computed here which is more conservative than the *RR* in Table two of the study (*RR* = 2.1, *95% CI*: 1.0-4.5).

^5^The STAI A scores reported separately for girls and boys were combined into one score in each of the three groups- non-users, users, and users with CUD using the formulae shown in the Additional file 1. The *ORs* in this study were computed based on these combined scores since all other studies in the current analysis reported anxiety scores in both genders combined rather than separately.

^6^The *ORs* in studies utilising VAHCS data from waves 1–8 were combined according to the formulae for combining dependent effect sizes shown in the Additional file 1.

^7^The *OR* was computed based on the following *N* of cases in the ‘Cannabis use at age 15’ and ‘Mental disorder- Internal (anxiety and depression)’ columns reported in Table five of the article: *N* = 20 (use/internal), *N* = 62 (use/no disorder), *N* = 84 (no use/internal), *N* = 637 (no use/no disorder).

*The standardised mean difference (Cohen’s *d*) was computed for user – non-user or CUD – non-user groups in all studies in Part 2 of this table. This effect size was then converted into *OR* using the formulae shown in the Additional file 1.

### Meta-analysis

The mathematical details of meta-analysis used in the current study are explained in the Additional file 1. The analysis was conducted on the Comprehensive Meta-Analysis software (CMA 2.2; Biostat, USA) using the random-effects model [57]. This model was chosen because it was expected that only a random sample of all studies (published or unpublished) on the topic was located during the systematic literature search, that the effect sizes would differ due to methodological heterogeneity among studies in this analysis, and that the current results could be generalisable to a wider general population.

In the first step of the analysis, a common effect size, the odds ratio (*OR*) and its 95% confidence interval (*95% CI*), were either computed by the authors or extracted from the *N* = 31 studies (Table 3). Subsequently, all effect sizes were weighted based on the inverse-variance method also known as the method of moments or DerSimonian and Laird method [58]. According to this method the weight is defined as the inverse of the sum of within-study and between-study variance. Compared to larger studies, the smaller-*N* studies usually have high variability of scores (high variance, low precision) and thus low weight. Consequently, such small studies have little influence on the overall mean weighted *OR* and *vice versa*.

The overall mean weighted *OR* was computed by dividing the sum of the product of weights and log *ORs* in each study by the sum of all weights. An *OR* < 1 indicated that cannabis use (or CUD) is associated with *lower* prevalence of anxiety and *vice versa*. The magnitude of *OR* was interpreted as follows [59]:

● *OR* > 1: 1.5 (small), 2.5 (moderate), ≥4 (large)

● *OR* < 1: 0.67 (small), 0.4 (moderate), ≤0.25 (large)

● *OR* = 1 and/or *95% CI* crossing the line of no effect (*OR* = 1): no association between cannabis use (or CUD) and anxiety.

The heterogeneity among studies was investigated using a *Q*-statistic and an *I*^*2*^ index [60]. The *Q*-statistic tests the null-hypothesis that *Q* = 0 meaning that there is homogeneity in effect sizes among studies included in the analysis. The *I*^*2*^ index expresses the *Q*-statistic on a 0-100% scale (*I*^*2*^ = 100% × (*Q*-*df*)/*Q*) using *df* = *k*-1 (*k* = number of studies) and can be interpreted as the variability in effect sizes due to real differences among studies (as opposed to differences due to chance alone). The interpretation criteria for *I*^*2*^ are: 25% (little heterogeneity), 50% (moderate heterogeneity), and 75% (high heterogeneity) [60].

Three separate meta-analyses were performed on the data reported in Table 3:

● anxiety/no anxiety vs. cannabis use/no use,

● anxiety/no anxiety vs. CUD/no CUD (or no use),

● anxiety + depression/no diagnosis vs. cannabis use/no use.

### Sensitivity and moderator analyses

The stability of the overall mean weighted *OR* in each analysis was investigated using one-study removed and cumulative analyses. These analyses show how the overall mean weighted *OR* changes if one study at a time is removed from all other studies or studies are added cumulatively over time. The moderator analyses (univariate meta-regression and subgroup analysis) were used to investigate the direction of the relationship between anxiety and cannabis use and the influence of systematic differences among studies on the overall mean weighted *OR*.

### Publication bias analyses

Publication bias refers to an overestimation of the overall mean weighted effect size in meta-analysis due to inclusion of studies based on large sample sizes and/or large effect sizes [57]. Such studies are more likely to be published and thus are easier to locate during a systematic literature search. In contrast, studies with smaller samples and/or small (often not statistically significant) effect sizes are either not published at all or published in smaller (often non-English language) journals that are not listed on major databases [57].

Publication bias in the current study was assessed using methods available in CMA, which are described in detail in the Additional file 1. The theoretical number of null-studies (with *OR* = 1) required to remove the statistical significance of the overall mean weighted *OR* was computed using Rosenthal’s Fail-Safe *N*[61]. Furthermore, the symmetry in a funnel plot of *OR* (on a logarithmic scale) vs. *SEM*[62] was assessed using the Duval and Tweedie’s Trim-and-Fill analysis [63]. Finally, the Begg and Mazumdar Rank Order Correlation (Kendall’s *tau b*) [64] and Egger’s regression [65] were used to investigate the relationship between the standardised *OR* and *SEM* or 1/*SEM* in each study*,* respectively. According to these methods, a systematic publication bias might be present in meta-analysis if the Fail-Safe *N* is small and smaller studies differ systematically (significantly) from the larger studies (funnel plot asymmetrical, correlation statistically significant, and regression intercept significantly different from zero) [57].

## Results

According to three separate meta-analyses summarised in Table 4, there was a small positive association between

**Figure 2:**
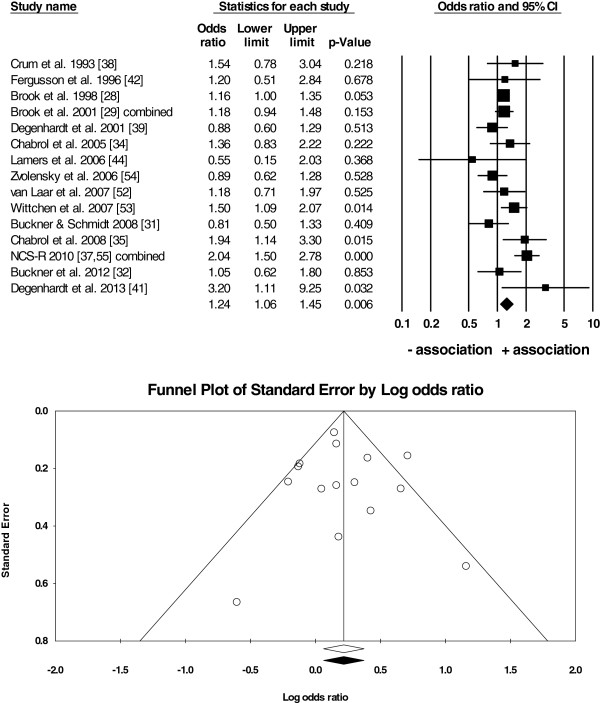
**Random-effects meta-analysis of*N*** **= 15 studies on anxiety vs. cannabis use/no use.** Notes: The forest plot (top) shows the effect size (*OR*) in each study, the weight of each study (size of the box), and the *95% CI* (the horizontal line through each box). The overall mean weighted *OR* is depicted as the centre of the diamond and its horizontal edges are the *95% CI*. Since the diamond did not cross the line of no effect (*OR*=1), there was an overall positive association between anxiety and cannabis use in *N*=15 studies (overall mean weighted *OR*=1.24, *95% CI*: 1.06-1.45). The funnel plot (bottom) shows the distribution of the individual effect sizes around the overall mean weighted *OR* (unfilled diamond). The trim-and-fill analysis revealed that the plot was symmetrical (the recomputed overall mean weighted *OR* depicted as the filled diamond overlaps with the unfilled diamond) suggesting that there was little evidence for a publication bias in the current analysis.

**Figure 3:**
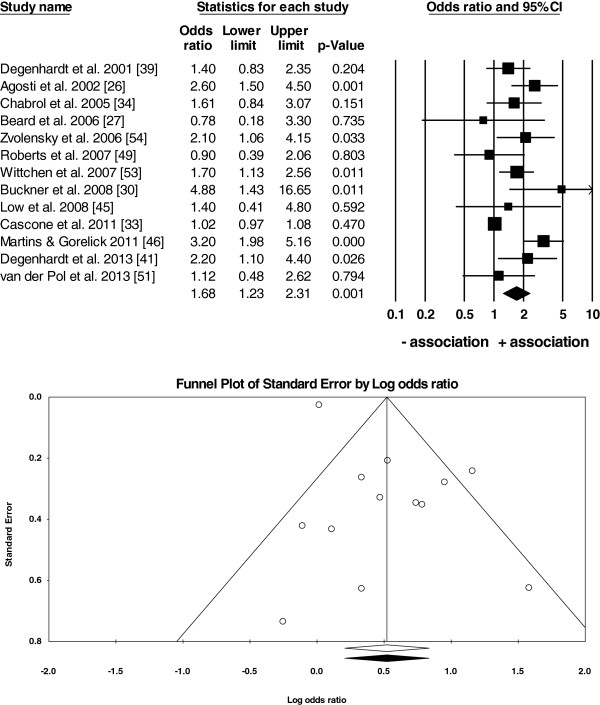
**Random-effects meta-analysis of*N*** **= 13 studies on anxiety vs. cannabis use disorder (CUD)/no CUD (or no use).** Notes: The forest plot (top) shows that there was an overall positive association between anxiety and CUD in *N*=13 studies (overall mean weighted *OR*=1.68, *95% CI*: 1.23-2.31). The funnel plot (bottom) was symmetrical suggesting that there was little evidence for a publication bias in the current analysis.

**Figure 4:**
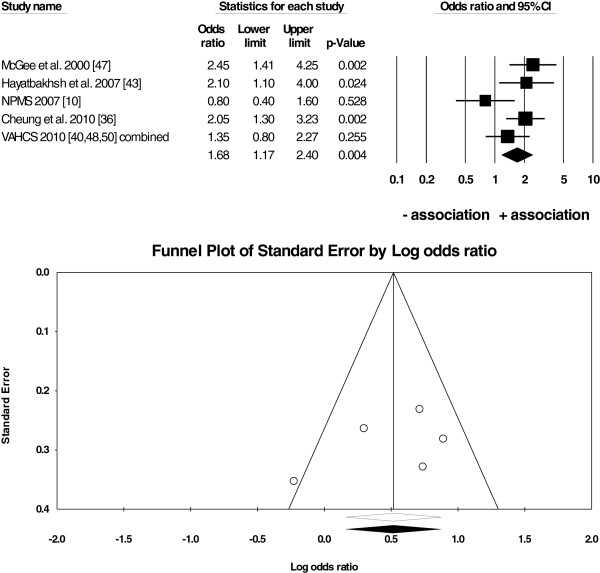
**Random-effects meta-analysis of*N*** **= 5 studies on anxiety + depression vs. cannabis use/no use.** Notes: The forest plot (top) shows that there was an overall positive association between anxiety+depression and cannabis use in *N*=5 studies (overall mean weighted *OR*=1.68, *95% CI*: 1.17-2.40). The funnel plot (bottom) was symmetrical suggesting that there was little evidence for a publication bias in the current analysis.

● anxiety and cannabis use (*OR* = 1.24, *95% CI*: 1.06-1.45; Figure 2),

● anxiety and cannabis use disorders (CUD; *OR* = 1.68, *95% CI*: 1.23-2.31; Figure 3), and

● anxiety + depression and cannabis use (*OR* = 1.68, *95% CI*: 1.17-2.40; Figure 4).

**Table 4:** Results of three random-effects meta-analyses on the association between anxiety vs. cannabis use or CUD and anxiety + depression vs. cannabis use

		**Anxiety vs. use/no use** ***N *** **= 15 (Figure**2**)**	**Anxiety vs. CUD/no CUD (or no use)** ***N *** **= 13 (Figure**3**)**	**Anxiety + depression vs. use/no use** ***N *** **= 5 (Figure**4**)**
**Overall mean weighted** ***OR***	*OR* (*95% CI*) *p*_two-tailed_	1.24 (1.06-1.45) .006*	1.68 (1.23-2.31) .001*	1.68 (1.17-2.40) .004*
**Heterogeneity statistics**	*Q(df)p*_two-tailed_*I*^*2*^	30 (14) .009* 53%	55 (12) <.0001* 78%	8 (4) .091 50%
**One-study removed analysis (Additional file****1****: Figure S1)**	Which studies, removed one at a time from the analysis, remove the significance of the overall mean weighted *OR*?	None	None	Cheung *et al.,* 2010 [36]
**Cumulative analysis (1993 to 2013) (Additional file****1****: Figure S2)**	Which studies, added to all previous studies one at a time, remove the significance of the overall mean weighted *OR*?	Crum *et al.*, 1993 [38]; Fergusson *et al*., 1996 [42]	Degenhardt *et al.*, 2001 [39]	NPMS, 2007 [10]
**Rosenthal’s Fail-safe** ***N *** **for** ***p > .05***	*N* studies needed to remove the significance of the overall mean weighted *OR* (*N* studies missing for every study in meta-analysis needed to remove the significance of the overall mean weighted *OR)*	*N*=49 (49/15 = 3)	*N*=105 (105/13 = 8)	*N*=17 (17/5 = 3)
**Duval and Tweedie’s Trim-and-Fill analysis**	Funnel plot symmetrical? *N* studies missing on either side of the overall mean weighted *OR*	YES	YES	YES
		*N*=0	*N*=0	*N*=0
**Begg and Mazumdar Rank Order Correlation**	*τ*	.13	-.19	-.30
	*p*_two-tailed_	.488	.360	.462
**Egger’s regression**	intercept	.25	1.73	−4.05
	*p*_two-tailed_	.748	.005*	.415

Note: Figure numbers refer to forest plots and funnel plots for each analysis (Figures S1 and S2 can be found in the Additional file 1).

*Abbreviations:CUD* cannabis use disorder (cannabis dependence and/or abuse/harmful use), *NPMS* the British National Psychiatric Morbidity Survey, UK; τ = Kendall’s correlation coefficient *tau b* with continuity correction.

*p < .05.

Thus, those with various anxiety disorders and concurrent anxiety + depression were more likely to use cannabis or had a CUD (dependence and/or abuse/harmful use) compared to those without anxiety disorders.

There was little evidence for a systematic publication bias because Fail-Safe *Ns* were high (*N* = 17 to 105), between 3 to 8 unpublished studies were needed for every study in the analysis to reduce the overall mean weighted *OR* to 1 (Table 4), and the funnel plots were symmetrical around the overall mean weighted *OR* (Figures 2, 3 and 4).

One-study removed analysis showed that the results of two analyses (anxiety vs. cannabis use and anxiety vs. CUD) were stable and not dependent on any one study alone (Table 4). Specifically, no study was able to eliminate the significant associations when removed from the analysis one at a time (Additional file 1: Figure S1). However, the results of the anxiety + depression vs. cannabis use analysis were dependent on one study [36]. Without this study there was only a non-significant trend (*p* = .058) towards a positive association between anxiety + depression and cannabis use (Table 4; Additional file 1: Figure S1).

The cumulative analysis showed that the significant and positive association between anxiety and cannabis use emerged as studies published in 1998–2013 were added to the overall analysis one at a time (Table 4). The results indicate that the overall effect size was always small as new studies were added to all previous studies (Additional file 1: Figure S2). Similar conclusion can be drawn regarding the small, positive, and significant association between anxiety and CUD that emerged in studies published in 2002–2013 and remained consistently small. In contrast, adding the results of the NPMS study [10] either eliminated or reduced the positive association between anxiety + depression vs. cannabis use in studies published in 2000–2010 (Additional file 1: Figure S2).

As expected there was a moderate-high heterogeneity among the effect sizes in the three meta-analyses (Table 4). Specifically, according to the *I*^*2*^ index, between 50-78% of variability in *ORs* was due to real differences among studies rather than chance. The inspection of study characteristics in Tables 2 and 3 revealed that such heterogeneity was probably due to two hypothesised systematic differences among studies:

(1) *OR* adjustment- in some studies *ORs* were controlled for confounders, such as other substance use, other substance use disorders, past anxiety, other psychiatric illness, and demographics (age, gender) while in other studies no statistical adjustment of *ORs* was used,

(2) year of publication- more recent studies might have applied more complex statistical modelling techniques to adjust *ORs* compared to the older studies regardless of when the data were actually collected.

One additional systematic difference among studies emerged only after the data were extracted from all studies (Tables 2 and 3):

(3) diagnosis of anxiety- in some studies cases had clinical diagnoses (DSM/ICD-based) while in other studies only the severity of anxiety scores on standardised instruments were reported (these studies were classified as having ‘non-clinical diagnoses’ for the purposes of the current analysis).

Taking into account these differences among studies, two types of moderator analyses were performed: subgroup analyses to compare the overall mean weighted *ORs* in subgroups of studies above and a univariate meta-regression to investigate if the year of publication could predict weighted *ORs* in all studies. Moderator analyses were not performed on studies with anxiety + depression because there was only one such study with unadjusted *OR* and one study with a clinical diagnosis of anxiety + depression.

According to the subgroup analyses, the majority of studies included in the current meta-analysis reported *ORs* adjusted for potential confounders (Table 5). Interestingly, the small positive association between anxiety and cannabis use (or CUD) was still present even after the adjustment for such confounders (Table 5; Additional file 1: Figure S3). Therefore, it appears that the higher odds for anxiety in cannabis users (with or without CUD) are not exclusively due to the effects of other substances, psychiatric illnesses, or demographics.

**Table 5:** Results of the moderator analyses (subgroup-analyses and univariate meta-regression)

	**Subgroup of studies**	**Anxiety vs. cannabis use/no use**			**Anxiety vs. CUD/no CUD (or no use)**		
		***N*** **studies**	***OR*** **(*95% CI *)**	***p***_**two-tailed**_	***N*** **studies**	***OR*** **(*95% CI *)**	***p***_**two-tailed**_
**Overall mean weighted** ***OR***		15	1.24 (1.06-1.45)	.006*	13	1.68 (1.23-2.31)	.001*
**Subgroup analyses** (Figure S3)							
*ORs* adjusted for confounders	Yes	12	1.24 (1.04-1.47)	.014*	10	1.66 (1.17-2.37)	.005*
	No	3	1.28 (.78-2.08)	.326	3	1.86 (1.10-3.15)	.021*
	Yes vs. No		*Q*(1) = .04; *p* = .848			*Q*(1) = 8.96; *p* = .003*	
Clinical diagnosis of anxiety (based on DSM/ICD)	Yes	9	1.29 (1.04-1.61)	.021*	11	1.87 (1.43-2.44)	<.001*
	No	6	1.17 (.93-1.48)	.186	2	1.14 (.78-1.66)	.509
	Yes vs. No		*Q*(1) = .30; *p* = .586			*Q*(1) = 36.91; *p* < .001*	
**Meta-regression**		***N*** **studies**	**slope**	***slope p***_**two-tailed**_	***N*** **studies**	**slope**	***slope p***_**two-tailed**_
Predictor: Year of publication Outcome: weighted *OR*							
		15	.02	.299	13	-.005	.901

Note: Confounders were: other substance use and/or other substance use disorders and/or past AD and/or other psychiatric illnesses and/or demographics. The subgroup analyses were conducted using the so-called mixed-effects model of meta-analysis [57]. According to this model the studies within each subgroup were combined using the random-effects model. However, since the number of subgroups was fixed (rather than randomly selected out of many subgroups), the overall mean weighted *ORs* in both groups were compared statistically using the between-groups *Q*-statistic based on the fixed-effect model of meta-analysis with *df* = 1 (number of subgroups-1). This approach to comparing *ORs* in independent subgroups of studies is equivalent to the independent samples *t*-test. Figure S3 is located in the Additional file 1.

*Abbreviations:AD* anxiety disorder, *CUD* cannabis use disorder (cannabis dependence and/or abuse/harmful use), *df* degrees of freedom, *OR* odds ratio.

*p < .05.

There were too few studies (*N* = 3) to reliably investigate these associations in studies with *ORs* unadjusted for confounders. There was a trend for a positive association between anxiety vs. CUD in *N* = 3 studies with unadjusted *ORs* (Table 5; Additional file 1: Figure S3).

Furthermore, the majority of current studies included cases with clinical diagnoses of anxiety according to DSM and/or ICD diagnostic systems. The small positive association between anxiety and cannabis use (or CUD) was present in the subgroups of studies with clinical diagnoses of anxiety (Table 5; Additional file 1: Figure S3). Thus, cannabis users with or without CUD have higher odds of clinically-relevant anxiety symptoms. There was a trend towards lack of such associations in the small subgroups of studies without clinical diagnoses of anxiety (Table 5; Additional file 1: Figure S3). Furthermore, according to the meta-regression, the weighted *ORs* were not univariately associated with the year of publication in studies included in the current analysis (Table 5).

**Figure 5:**
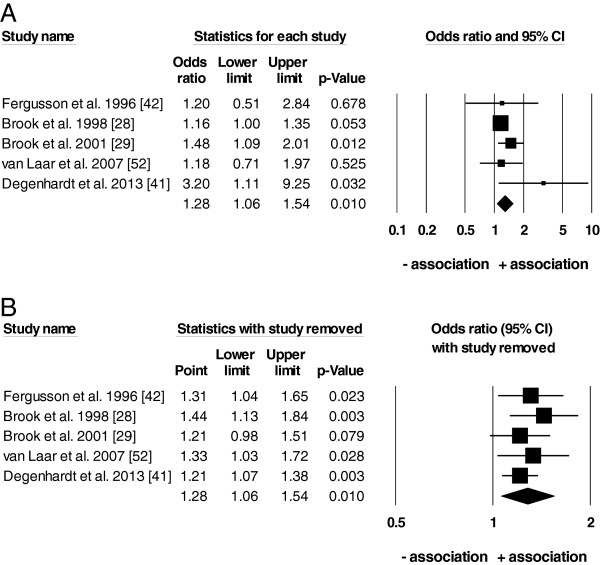
**Random-effects meta-analysis (A) and one-study removed analysis (B) of*N*** **= 5 studies on cannabis use at baseline and anxiety at follow-up (all*ORs*adjusted for potential confounders).** Notes: The top forest plot **(A)** shows that there was an overall positive association between cannabis use at baseline and anxiety at follow-up in *N*=5 studies according to the random-effects meta-analysis (overall mean weighted *OR*=1.28, *95% CI*: 1.06-1.54). The bottom forest plot **(B)** shows the outcome of the one-study removed sensitivity analysis. ‘Point’ on plot B refers to the overall mean weighted *OR* of all studies without the study in each row. This analysis revealed that the positive association between cannabis use at baseline and anxiety at follow-up was still present when the study with the largest *OR* in plot A (*OR*=3.20) was removed from the analysis.

Finally, even though a positive association does not provide evidence for causation, it was possible to investigate the temporal relationship between anxiety and cannabis use in a small subset of *N* = 5 studies that reported *ORs* for cannabis use at baseline and anxiety at follow-up. This analysis showed that a cohort of those using cannabis at baseline was significantly more likely to have symptoms of anxiety at follow-up in studies adjusted for confounders (*OR* = 1.28, *95% CI*: 1.06-1.54, *p* = .01; *N* = 5 studies; Figure 5A). The opposite relationship was investigated in only one study [29]. The results showed that there was no association between anxiety at baseline and a regular cannabis use at follow-up (*OR* = .94, *95% CI*: .86-1.03; Table 3). Interestingly, the one-study removed analysis (Figure 5B) showed that the positive association between cannabis use at baseline and anxiety at follow-up was not entirely due to the study by Degenhardt and colleagues [41] with the largest effect size (*OR* = 3.20) in the analysis of *N* = 5 studies. However, the exclusion of the study by Brook and colleagues [29] removed the traditional level of statistical significance from the association, most likely due the low statistical power of the analysis of *N* = 4 studies only. The inspection of the effect sizes alone suggests that there was a trend towards a consistently small and positive relationship between cannabis use at baseline and anxiety at follow-up (*OR* range: 1.21-1.44) after the removal of each of the five studies, one at a time (Figure 5B).

## Discussion

The current study is the first meta-analysis to quantitatively describe the relationship between anxiety and cannabis use using data from *N* = 31 studies on samples drawn from approximately 112,000 cases from the general population of 10 countries (Australia, Canada, Columbia, France, Germany, Netherlands, New Zealand, Switzerland, UK, USA). The main finding of the current meta-analysis is that cohorts with anxiety are more likely to use cannabis (odds of 1.24) or have a cannabis use disorder (odds of 1.68; Table 4). Similarly, cohorts with concurrent anxiety and depression are also more likely to use cannabis (odds of 1.68; Table 4). These findings are based on samples from the general population neither in treatment for anxiety nor cannabis use disorders. The current study quantitatively supplements the findings of one other systematic review on the relationship between anxiety and cannabis use in *N* = 7 studies [10].

It can only be speculated that the associations above would be higher in samples perceiving their anxiety and/or cannabis use as problematic and thus seeking professional treatment for either one or both of these conditions. In support of this statement, our results show a trend towards a higher and more stable relationship between anxiety and cannabis use disorders that might eventually require treatment (*OR* = 1.68, *95% CI*: 1.23-2.31), than cannabis use alone (*OR* = 1.24, *95% CI*: 1.06-1.45; Table 4). Similarly, higher rates of comorbidity would be expected in vulnerable populations often not included in the population-based surveys due to being homeless, imprisoned, or inpatient in psychiatric or rehabilitation institutions [66].

All three overall effect sizes were only small and their *95% CI* were close to the line of no effect (*OR =* 1) in the current study (Table 4). Such small *ORs* were probably due to the heterogeneous duration and definition of ‘anxiety disorders’ and ‘cannabis use/CUD’ used in the current analysis. In general, the duration of diagnoses and/or cannabis use/CUD ranged from within past 12 months to lifetime (Table 2). Furthermore, some studies investigated a wide range of anxiety diagnoses according to DSM/ICD criteria, while others focused on ‘narrow’ diagnoses, such as PTSD or OCD, only. Similarly, the definition of ‘cannabis use’ ranged between ‘use >1× (lifetime)’ to ‘use > daily (last 6 months)’ and the prevalence of particularly heavy cannabis use (>daily) was low (Table 2). Since the total lifetime duration of cannabis use was often not reported in studies, it was not possible to further investigate the differences in results among subgroups of studies with more homogenous definition, frequency, and/or duration of cannabis use.

It is interesting that the relationship between anxiety and cannabis use was still present and positive even after controlling for confounders (other substance use/psychiatric comorbidity/demographics) in studies with mostly non-frequent users without cannabis use disorders in the general population (Table 5). Furthermore, such positive association was found only in studies using cases with clinical symptoms of anxiety disorders (according to DSM/ICD criteria), but not in studies that measured severity of anxiety using standardised scales or symptom checklists (Table 5). It has been suggested that particularly a high-dose cannabis use in drug-naïve users could either induce some acute symptoms of anxiety (intense fear, panic attacks) without necessarily causing anxiety disorders or result from withdrawal in those with cannabis dependence [16]. Although we have not controlled for the acute cannabis use (dose, concentration of cannabinoids in urine, withdrawal symptoms), our results show that any level of cannabis use is positively related only to clinically relevant symptoms of anxiety. Thus, it is unlikely that the positive association between anxiety and cannabis use in the current study was due to exclusively acute (non-clinical) symptoms of anxiety induced by a heavy (acute) cannabis use.

In terms of direction, cannabis use could further exacerbate existing symptoms of anxiety depending on the genetic vulnerability, severity of anxiety symptoms, gender, and age, among other factors [16]. In fact, we have found that baseline cannabis use was indeed positively associated with anxiety at follow-up in *N* = 5 studies that reported *ORs* controlled for potential confounders and four of which used the clinical diagnoses of anxiety (Figure 5). Although it is tempting to state that cannabis use at baseline *caused* anxiety at follow-up (Figure 5), there is little direct evidence to support such a conclusion in the current study. The effect size of this relationship had little statistical stability in terms of only a small positive value (*OR =* 1.28) and the 95% confidence interval located close to the line of no effect (*OR* = 1). Furthermore, other relevant factors necessary to establish causality in epidemiological research, such as the time order, misclassification, and residual confounding [67,68], were not assessed in the current analysis. Specifically, our results are based on cohorts of cases with generally low proportions of (mostly low-level) cannabis users who could have changed their group membership over time due to other factors than cannabis use alone. For example, a non-user with no anxiety at baseline could have become a user (due to a one-off use) with a diagnosis of anxiety related to a specific event (such as PTSD resulting from a traffic accident) at follow-up. Another plausible alternative would be that some cases were misclassified because of the slow onset of some anxiety disorders or low severity of anxiety symptoms already present at baseline but insufficient for a formal clinical diagnosis until a later point in time (at follow-up). In general, a lack of longitudinal follow-up of the *same* cases makes it difficult to validly and reliably study the causation in the association between any mental illness and cannabis use. Furthermore, the individual *ORs* were adjusted for different potential confounders in the *N* = 5 primary studies (Table 3). This is of importance because our observed positive association might have resulted from (a) residual confounding due to inadequate controlling for potential confounders in primary studies as well as our analysis and/or from (b) multiple, unmeasured factors causally related to each other, that were not taken into account in both the primary studies and our analysis [67]. For example, we have used an unspecific and broad binary criterion to classify studies based on presence/absence of statistical adjustment of *OR* for *any* relevant confounders in our analysis (Table 5). However, it is likely that including specific confounders measured as scale variables would have been a more effective strategy to reduce their direct or indirect effect on our association. One obvious candidate for such a scale confounder could be the heaviness of cannabis use measured as frequency/day, total length of use (in years), and/or dose of cannabis/day. Other important confounders, possibly causally related to heaviness of cannabis use, could include age, severity of comorbid psychiatric diagnoses, and use of other substances. However, it must be stressed that meta-analysis utilises effect sizes based on group data from primary studies. Thus adjustments for the *same* (multiple) potential confounders would need to be applied in *all* primary studies using individual case data to be systematically carried over to a meta-analysis. The only sensitivity analysis that we were able to conduct on such a small number of studies included in our analysis (*N* = 5) was the one-study removed analysis (Figure 5B). This analysis showed that there was a trend towards a positive (but small) association between cannabis use at baseline and anxiety at follow-up even after the removal of the study with the largest effect size of *OR* = 3.20 [41] from this analysis. Therefore, regardless of causation, it appears that the relationship between cannabis use at baseline and anxiety at follow-up is (at most) only small.

Due to lack of data we were unable to investigate the relationship between anxiety at baseline and cannabis use at follow-up. Presence of such a (positive) relationship could be used to support the self-medication hypothesis suggesting that those with anxiety disorders could use cannabis to relax and better cope with stress [16]. The self-medication hypothesis would also need to be investigated taking into account the difference between the acute and long-term effects of cannabis use and the frequency/dose/total duration of cannabis use. In general, it appears that cannabis has biphasic or bidirectional effect on anxiety [18]. Thus, those with anxiety could experience some acute relief from their symptoms after low-frequency and low-dose cannabis use. However, a regular and heavier use could lead to development of cannabis use disorders and, in turn, be associated with worsening of anxiety symptoms. These biphasic relationships could result from a dose-dependent interaction between the active ingredient of cannabis (delta-9-tetrahydrocannabinol) and (dysregulation of) endocannabinoid and neurotransmitter systems, including dopamine, GABA, glutamate, serotonin, and noradrenaline [16,18]. Current results suggest that particularly cannabis use disorders (likely resulting from heavy use) are related to anxiety (Table 4). Similarly, dependent frequent cannabis users, but not the frequent users without dependence, had more anxiety disorders compared to the general population [51]. Furthermore, the age of initial cannabis use and/or development of cannabis use disorders and anxiety symptoms might be a necessary factor to control for when testing the association between anxiety and cannabis use. For example, early cannabis use may affect the adolescent neuromaturation and cognitive functioning [69] and thus predispose the users to subsequent development of anxiety and other mental health problems [23]. However, genetic vulnerability to anxiety could also contribute to poor early social and cognitive functioning, limited educational and employment prospects, and subsequent onset of substance use [16].

The current results could have some implications for policy making purposes. An association of a small magnitude between anxiety and cannabis use supports the argument that compared to the impact of other illicit drugs, alcohol, or tobacco, the sole role of cannabis use in public health could be judged as only modest [24] and cannabis use is only a minor contributor to the overall disease burden worldwide [8]. Thus, the current results do not provide a strong evidence for prohibiting cannabis. However, because the two conditions co-exist at a rate higher than statistical chance alone and, since cannabis use and anxiety disorders are common particularly among the younger generations, governments should focus on providing affordable, easily accessible, and effective mental health care. According to the World Mental Health (WMH) Surveys, mental disorders, including anxiety and substance abuse, are a crucial societal issue because they contribute to a loss of ‘healthy life years’ in terms of reduced working capacity and the required treatment [70]. Such a burden should be particularly addressed in times of increased daily stress and financial insecurity that are likely to contribute to development of some anxiety disorders. Major financial problems were also identified as more important predictors of development of cannabis dependence than other ‘stable’ vulnerability factors (such as childhood adversity, own or family history of mental illness or problematic substance use, and personality) [71].

It is difficult to effectively treat comorbidity and to quantify the recovery from such a comorbidity to a meaningful life [66]. In general, patients with dual diagnoses (problematic substance use and mental health comorbidity) are more satisfied with integrated comorbidity treatment than with standard treatment without explicit focus on both diagnoses [72]. Detecting and addressing cannabis use in patients presenting with anxiety disorders might also be important because cannabinoids could theoretically interact with any pharmacological treatment for anxiety. However, the accuracy of self-reported cannabis use depends on appropriate conditions of data collection, including confidentiality and anonymity [73]. Such conditions could in turn improve the accuracy of psychiatric diagnosis, and contribute to more patients receiving treatment for substance use disorders in the clinical practice [74]. Since particularly the infrequent users rarely seek professional help, it might be useful to include anxiety as one risk associated with cannabis use in educational programs on the level of schools, community health centres, and in the context of primary care settings [10,40]. Especially the motivational interviewing and motivational enhancement techniques might be useful for approaching cannabis use disorders because young people often lack the motivation to address their substance use and thus miss early intervention strategies [33,50]. Reducing social stigma and providing informal care could also contribute to more problematic cannabis users (with cannabis use disorders) subsequently seeking professional treatment [75]. Furthermore, recovery from comorbidity might be enhanced by using social networks and peer education and support [66]. While people with comorbidity have less supportive social networks than those with problematic substance use alone, such networks could still be utilised as important communication channels particularly in the vulnerable populations [66].

The current study has a number of strengths. First, the assessment of suitability of studies for the analysis, deriving the extraction rules, and the data extraction were done independently by two authors to reduce bias in the data. This issue was particularly important because some studies reported multiple estimates of *ORs*. Our procedure was also consistent with the data extraction from *N* = 7 studies done by Moore and colleagues [10] (according to Figure six of their study) that were included in our analysis. Second, the data in both meta-analyses come from large-*N*, longitudinal surveys based on representative samples from the general population in 10 countries. Our study was more inclusive by also extracting data from small-*N*, cross-sectional studies. This approach improved the power of our analysis and allowed us to perform three separate overall analyses and subsequent subgroup analyses on more homogenous subsets of studies in contrast to Moore *et al.*[10] who were unable to meta-analyse the results of highly heterogeneous *N* = 7 studies. While small-*N* studies can be considered unrepresentative and of poor quality compared to large longitudinal studies, some of the former included carefully screened participants and excluded other substance users and those with other psychiatric conditions. Therefore, the samples in these smaller studies were not necessarily of ‘worse quality’ than those in large-scale surveys, where comorbidity with other substance use/psychiatric illness was often present and only controlled for statistically rather than by excluding such participants from further analyses. In addition, the prevalence of cannabis use and/or anxiety was low in the large longitudinal studies and, thus, the *ORs* in these studies were also computed based on small *Ns*. Third, the studies selected for the current analysis were performed in a number of countries reducing the bias in the results towards any one country and/or one research team only. Although both authors of the current study are multilingual, the search for studies was conducted in English only because most of the largest studies on the topic are conducted in the English-speaking, western world (possibly due to high costs of longitudinal studies) and/or are published in English. Consequently, even if searching in other languages, finding of non-English studies is more difficult because they are often not listed on the largest scholarly databases. Finally, a systematic assessment of publication bias with a number of tests (funnel plots in Figures 2, 3, 4 and results in Table 4) suggests that there was little evidence for such a bias in the current analysis. Specifically, our meta-analysis included studies with either positive, negative, or lack of associations between anxiety and cannabis use/cannabis use disorders rather than studies reporting only strong and positive associations.

An important limitation of the current study is the literature search strategy. While Moore *et al.*[10] searched a large number of databases, the current search was performed on two databases only (Table 1). The rationale behind this search strategy was that we expected to find the most important sources on Medline and PsycInfo databases. These two databases were also the most-relevant to the topic databases available at our institution. Our search strategy had failed to find *N* = 5 published studies from Figure six in Moore *et al*. [10]. While this fact could be considered as clear evidence for a publication bias (or at least a poor search strategy), none of the *N* = 5 studies specifically addressed anxiety *and* cannabis use (a combination of our search terms; Table 1). Instead, the titles of these studies included *substance use* and anxiety, cannabis use and *mental health*, or *substance use* and *mental health*. Furthermore, both meta-analyses had failed to electronically locate a large longitudinal study from the USA [26] even though the title contained the relevant search terms. This study was obtained from our hand searches but was omitted from Moore *et al.*[10] (even though it met the inclusion criteria of that study). Therefore, it is unlikely that any meta-analysis would include a complete set of studies on this topic (both published and unpublished) because, if published, they might not be listed on major scholarly databases, listed using non-specific terms only (such as substance use and mental illness), and access to all print material in all countries is hardly possible. One reasonable compromise is to conclude that our results cannot be extrapolated to the general population (of the whole world) but rather apply to countries and samples used in the current study alone. According to Table 2, if the same studies are counted only once and the latest wave is used as the total sample size/study, then the current results apply to samples drawn from approximately 112,000 cases from the general population of 10 countries.

## Conclusions

The results of the current meta-analysis suggest that anxiety is positively related to cannabis use or cannabis use disorders in cohorts, not in treatment for anxiety or cannabis use disorders, drawn from the general population of 10 countries. These associations were only small in magnitude, but were observed even after controlling for confounding factors (demographics, other substance use, and/or other psychiatric comorbidity). The prospective analysis revealed that cannabis use at baseline was positively associated with anxiety at follow-up. While the causal direction of this relationship could not be established using the available data, this result suggests that even infrequent cannabis use is associated with clinically relevant symptoms of anxiety. Therefore, an appropriate assessment of cannabis use might be necessary for an effective treatment of anxiety disorders.

## Abbreviations

AD: Anxiety disorder; ADAD: Adolescent Drug Abuse Diagnosis (based on Addiction Severity Index); AMD: Anxiety + depression; AP: Agoraphobia; AUDADIS: Alcohol Use Disorders and Associated Disabilities Interview Schedule; BAI: Beck Anxiety Inventory; CA: Cannabis abuse; CAMH: Centre for Addiction and Mental Health Monitor survey, Canada; CanDep: The Dutch Cannabis Dependence Study, Netherlands; CD: Cannabis dependence; CHDS: Christchurch Health and Development Study, New Zealand; CIDI: Composite International Diagnostic Interview; CIS-R: Clinical Interview Schedule- Revised; CSHS: Colorado Social Health Survey, USA; CUD: Cannabis use disorder (abuse/harmful use and/or dependence); D + : Frequent cannabis users with dependence in CanDep study; DIS: Diagnostic Interview Schedule; DISC: Diagnostic Interview Schedule for Children; DMHDS: Dunedin Multidisciplinary Health and Development Study, Dunedin, New Zealand; ECA: Epidemiological Catchment Area program, USA; EDSP: Early Developmental Stages of Psychopathology study, Germany; GAD: generalized anxiety disorder; GHQ-12: General Health Questionnaire (12 items); HSC: Hopkins Symptom Checklist; K-SADS: Schedule for Affective Disorders and Schizophrenia for School-Age Children; LIFE: Longitudinal Interval Follow-up Evaluation; MUSP: Mater University Study of Pregnancy, Brisbane, Australia; NCS: National Comorbidity Survey, USA; NCS-R: National Comorbidity Survey- Replication, USA; NEMESIS/NEMESIS-2: Netherlands Mental Health Survey and Incidence Study (study 1: 1996–1999 and study 2: 2007–2009); N2: NEMESIS-2 cases Netherlands; NESARC: National Epidemiological Survey on Alcohol and Related Conditions, USA; NoRMHS: The Northern Rivers Mental Health Study, New South Wales, Australia; NPMS: The British National Psychiatric Morbidity Survey, UK; NSMHWB: National Survey of Mental Health and Well-Being, all states, Australia; OAD: Overanxious disorder; OCD: Obsessive compulsive disorder; PA: Panic attacks; PD: Panic disorder; PRIME-MD: Primary Care Evaluation of Mental Disorders; PTSD: Post-traumatic stress disorder; SA: Separation anxiety; SAD: Social anxiety disorder/social phobia; SCID-I/NP: Structured Clinical Interview for DSM-IV, non-patient version; SIAS: Social Interaction Anxiety Scale; SP: Specific phobias; STAI: State-Trait Anxiety Inventory; STAI-Y: STAI for Youth; STAI-Y A: STAI state anxiety subscale; STAI-Y B: STAI trait anxiety subscale; T: Specific wave of data collection in longitudinal studies; TH2K: Teen Health 2000 Study, Houston, USA; VAHCS: Victorian Adolescent Health Cohort Study, Victoria, Australia; YASR: Young Adult Self-Report.

## Competing interests

Both authors have no competing interests. There was no external funding for this study.

## Authors’ contributions

Both authors have equally contributed to this study: LTL conducted the systematic search, extracted data from a pilot sample of *N* = 17 studies, conducted preliminary analyses on these data; KKK extracted data from all studies, conducted the final analysis, and wrote the paper; both authors agreed on the data extraction procedure, double-checked the extracted data from all *N* = 31 studies, and critically revised the paper. Both authors read and approved the final manuscript.

## Authors’ information

Dr. Karina Kedzior completed her PhD in the clinical neurosciences at the University of Western Australia, Perth, Australia, in 2004. Her research so far has focused on the relationship between cannabis use and neurophysiological functioning (prepulse inhibition of the startle reflex) in cannabis users and schizophrenia patients. She has also investigated the validity of self-reports of cannabis and other substance use in cannabis users and schizophrenia patients, the validity of cannabis dependence diagnoses on the CIDI, and the association between cannabis use and depression. She is currently a Lecturer in Statistics and Research Methods at Jacobs University Bremen, Germany, where she teaches the traditional statistical methods (including logistic regression) and meta-analysis. She is a co-author of four other meta-analyses.

Lisa Laeber completed her BA in Integrated Social and Cognitive Psychology at Jacobs University Bremen, Germany, in June 2013. Throughout her degree she studied all methods necessary for performing of the current analysis (linear and logistic regressions, meta-analysis) and completed various courses on advanced statistics in psychology (structural equation modelling, mixed methods, and an ERASMUS intensive program on quantitative approaches to psychological processes). Her bachelor thesis on the relationship between anxiety and cannabis use in *N* = 17 studies submitted in April 2013 (under the supervision of Dr. Kedzior) was used as a pilot study for the purpose of this publication.

## Pre-publication history

The pre-publication history for this paper can be accessed here:

http://www.biomedcentral.com/1471-244X/14/136/prepub

## Supplementary Material

Additional file 1 Supplementary Appendix. Click here for file
