# Supplementary material for: A positive association between anxiety disorders and cannabis use or cannabis use disorders in the general population- a meta-analysis of 31 studies
Source: BMC Psychiatry. 2014 May 10;14:136. doi: 10.1186/1471-244X-14-136 (PMC4032500; doi:10.1186/1471-244X-14-136)
Supplement: Additional file 1 — Supplementary Appendix. [file 1471-244X-14-136-S1.pdf]

Additional file 1 (Supplementary Appendix)

Kedzior K, Laeber L: **A positive association between anxiety disorders and cannabis use or cannabis use disorders in the general population- a meta-analysis of 31 studies.** *BMC Psychiatry* 2014.

Contents

Computation of odds ratio (*OR*) and its 95% confidence interval (*95%CI*) 2

Computation of standardised mean difference (Cohen's *d*) and its conversion to *OR* 3

Combining of data in independent groups to compute *Cohen's d* 4

Combining *OR* from studies with dependent data (using the same cases/data sets) 5

Random-effects meta-analysis of *OR* (binary) data 6

Publication bias analyses in Comprehensive Meta-Analysis (CMA) software 9

Table S1. Exclusion criteria applied to *N*=267 studies 11

Table S2. Studies assessed in full length (*N*=49) and reasons for exclusion 12

Figure S1. Random-effects one-study removed analysis 14

Figure S2. Random-effects cumulative analysis 16

Figure S3. Random-effects subgroup analyses 18

Bibliography 20

The mathematical approach to meta-analysis in this document is based on Borenstein *et al.*, 2009 [1].

Computation of odds ratio (*OR*) and its 95% confidence interval (*95%CI*)

The odds ratios (*OR*) were not reported in some studies and were computed according to the following formulae based on the number of cases (letters A-D) in the following groups (‘cannabis user’ or ‘cannabis user with cannabis use disorder (CUD)’, ‘non-user’ or ‘no CUD’, ‘anxiety’ or ‘anxiety+depression’, ‘no anxiety’ or ‘no diagnosis’) below [1, Table 5.1, p. 33]:

	ANXIETY (or ANXIETY+DEPRESSION)	NO ANXIETY (or NO DIAGNOSIS)
CANNABIS USER (or CUD)	A	B
NON-USER (or NO CUD)	C	D

The *OR* for anxiety (vs. no anxiety) in cannabis users (vs. non-users or CUD vs. no CUD) was computed as follows [1, p. 36]:

$$OR = \frac{A/B}{C/D}$$

The *OR* for cannabis use (vs. no use or CUD vs. no CUD) in anxiety (vs. no anxiety) was computed as follows:

$$OR = \frac{A/C}{B/D} = \frac{A/B}{C/D}$$

The formulae indicate that both *ORs* are equivalent.

Since the *OR* is limited on its lower end (it cannot be negative) but can take on any positive value, its distribution is skewed [2]. Thus, to maintain symmetry and obtain an approximately normal distribution, the *95%CI* was computed based on the log (natural logarithmic, *ln*) scale using the values from the contingency table above as follows [2]:

$$LogOR = ln(OR)$$

$$LogLower\ (ln\ \text{lower bound}\ 95\%CI) = LogOR - (1.96 \times SE_{LogOR})$$

$$LogUpper\ (ln\ \text{upper bound}\ 95\%CI) = LogOR + (1.96 \times SE_{LogOR})$$

$$SE_{LogOR} = \sqrt{V_{LogOR}}$$

$$V_{LogOR} = \frac{1}{A} + \frac{1}{B} + \frac{1}{C} + \frac{1}{D}$$

In the final step the LogLower and LogUpper were converted back (antiloged) into *OR* scale as follows [2]:

$$\text{Lower}\ (95\%CI) = e^{LogLower}$$

$$\text{Upper}\ (95\%CI) = e^{LogUpper}$$

Computation of standardised mean difference (Cohen's *d*) and its conversion to *OR*

Some studies reported the severity of anxiety scores based on standardised scales in user and non-user (or CUD and no CUD) groups. Based on mean (*M*), standard deviation (*SD)* of scores and group size (*N)* in each group the standardised mean difference (Cohen's *d*) and its variance (V_d) were computed in these studies as follows [1, p. 26-27]:

$$d = \frac{M_{User} - M_{Non-user}}{SD_{pooled}}$$

$$SD_{pooled} = \sqrt{\frac{(N_{User} - 1)SD_{User}{}^2 + (N_{Non-user} - 1)SD_{Non-user}{}^2}{N_{User} + N_{Non-user} - 2}}$$

$$V_d = \frac{N_{User} + N_{Non-user}}{N_{User}N_{Non-user}} + \frac{d^2}{2(N_{User} + N_{Non-user})}$$

The *d* and V_d were converted into the log *OR* scale as follows [1, p. 47]:

$$LogOR = d \times \frac{\pi}{\sqrt{3}}$$

$$V_{LogOR} = V_d \times \frac{\pi^2}{3}$$

$$SE_{LogOR} = \sqrt{V_{LogOR}}$$

Finally, the log *95%CI* (LogLower and LogUpper) was computed and all log values were antiloged as follows [2]:

$$\text{LogLower}=LogOR - (1.96 \times SE_{LogOR})$$

$$\text{LogUpper}=LogOR + (1.96 \times SE_{LogOR})$$

$$OR= e^{LogOR}$$

$$\text{Lower } (95\%CI)= e^{LogLower}$$

$$\text{Upper } (95\%CI)= e^{LogUpper}$$

Combining of data in independent groups to compute *Cohen's d*

One study reported the severity of anxiety scores separately for boys and girls in non-user, user and user with CUD groups. Thus, the mean (*M*) and the standard deviation (*SD*) of scores for boys and girls had to be combined into a single score in each group (non-user, user and user with CUD).

The total sample size per group (N_{1+2}) was the sum of N_1 (number of boys) and N_2 (number of girls) in that group. The combined mean severity of anxiety score for boys (M_1) and girls (M_2) in each group (M_{1+2}) was computed as follows [1, p. 222]:

$$M_{1+2} = \frac{N_1 M_1 + N_2 M_2}{N_1 + N_2}$$

The combined standard deviation of the mean severity of anxiety scores for boys (SD_1) and girls (SD_2) in each group (SD_{1+2}) was computed as follows [1, p. 222]:

$$SD_{1+2} = \sqrt{\frac{(N_1 - 1)SD_1^2 + (N_2 - 1)SD_2^2 + \frac{N_1 N_2}{N_1 + N_2}(M_1 - M_2)^2}{N_1 + N_2 - 1}}$$

Based on the single *M*, *SD*, and *N* values per group, the standardised mean difference, Cohen's *d*, was computed for the difference between users – non-users and CUD – non-users as explained under subsection 2 of this document.

Combining *OR* from studies with dependent data (using the same cases/data sets)

If the same cases were used in different studies or same studies provided two estimates of *ORs* based on the same cases then a mean of such *ORs* and its variance were computed. Because the same cases were used it was assumed that the *ORs* were dependent and thus the correlation between them is r=1. This approach is conservative in that it overestimates the variance of the mean *OR* and thus increases the length of the *95%CI* [1, p. 232]. The two *ORs* and their *95%CIs* were first converted into a log scale as follows [1, p. 36, 3]:

$$LogOR_1=ln(OR_1)$$

$$LogOR_2=ln(OR_2)$$

$$\text{LogLower}_1 = ln(\text{lower bound } 95\%\ CI_1)$$

$$\text{LogUpper}_1 = ln(\text{upper bound } 95\%\ CI_1)$$

$$\text{LogLower}_2 = ln(\text{lower bound } 95\%\ CI_2)$$

$$\text{LogUpper}_2 = ln(\text{upper bound } 95\%\ CI_2)$$

Then, an arithmetic mean ($LogOR_{combined}$) of $LogOR_1$ and $LogOR_2$ was computed. Next, the variance of each *OR* was computed separately as follows [3]:

$$SE_{LogOR1} = [(\text{LogUpper}_1 - \text{LogLower}_1)/2]/1.96$$

$$V_{LogOR1} = (SE_{LogOR1})^2$$

$$SE_{LogOR2} = [(\text{LogUpper}_2 - \text{LogLower}_2)/2]/1.96$$

$$V_{LogOR2} = (SE_{LogOR2})^2$$

The two individual estimates of variance (V_{LogOR1} and V_{LogOR2}) for both *ORs* were combined into one variance estimate ($V_{LogORcombined}$) using r=1 as follows [1, p. 228]:

$$V_{LogORcombined} = \frac{V_{LogOR1} + V_{LogOR2} + 2r\sqrt{V_{LogOR1}}\sqrt{V_{LogOR2}}}{4}$$

$$SE_{LogORcombined} = \sqrt{V_{LogORcombined}}$$

Finally, the log *95%CI* of $LogOR_{combined}$ ($LogLower_{combined}$ and $LogUpper_{combined}$) was computed and all log values were antiloged as follows [2]:

$$\text{LogLower}_{\text{combined}} = LogOR_{combined} - (1.96 \times SE_{LogORcombined})$$

$$\text{LogUpper}_{\text{combined}} = LogOR_{combined} + (1.96 \times SE_{LogORcombined})$$

$$OR_{combined} = e^{LogORcombined}$$

$$\text{Lower } (95\%CI_{combined}) = e^{LogLowercombined}$$

$$\text{Upper } (95\%CI_{combined}) = e^{LogUppercombined}$$

Random-effects meta-analysis of *OR* (binary) data

All computations were done by converting each study's *OR* and its *95%CI* into the log scale [1, p. 36, 3]:

$$LogOR = ln(OR)$$

$$\text{LogLower} = ln(\text{lower bound } 95\%\ CI)$$

$$\text{LogUpper} = ln(\text{upper bound } 95\%\ CI)$$

Next, the within-study variance for each study was computed as follows [3]:

$$SE_{LogOR} = [(\text{LogUpper} - \text{LogLower})/2]/1.96$$

$$V_{LogOR}\ (\text{within-study variance}) = (SE_{LogOR})^2$$

The weight of each study (W_{LogOR}) was computed according to the random-effects model as follows [1, Chapter 12]:

$$W_{LogOR} = \frac{1}{V_{LogOR} + T^2},$$

where V_{LogOR} is the within-study variance of $LogOR$ and T^2 is the between-study variance which was computed according to the method of moments also known as the DerSimonian and Laird method [4] and using $df=k-1$ (k=number of studies) as follows:

$$T^2 = \frac{Q - df}{C}$$

$$C = \sum \frac{1}{V_{LogOR}} - \frac{\sum \left(\frac{1}{V_{LogOR}} \right)^2}{\sum \frac{1}{V_{LogOR}}}$$

$$Q = \sum \frac{LogOR^2}{V_{LogOR}} - \frac{\left(\sum \frac{LogOR}{V_{LogOR}} \right)^2}{\sum \frac{1}{V_{LogOR}}}$$

The overall mean weighted effect size (M_{LogOR}) of all studies and its variance (V_{MLogOR}) were computed as follows:

$$M_{LogOR} = \frac{\sum W_{LogOR} \times LogOR}{\sum W_{LogOR}}$$

$$V_{M_{LogOR}} = \frac{1}{\sum W_{LogOR}}$$

$$SE_{M_{LogOR}} = \sqrt{V_{M_{LogOR}}}$$

The lower and upper bounds of the log *95% CI* of M_{LogOR} ($LogLower_{MLogOR}$ and $LogUpper_{MLogOR}$) were computed as follows:

$$\text{LogLower}_{MLogOR} = LogOR_{MLogOR} - (1.96 \times SE_{MLogOR})$$

$$\text{LogUpper}_{MLogOR} = LogOR_{MLogOR} + (1.96 \times SE_{MLogOR})$$

Next, the *z*-score for M_{LogOR} was computed to test the null-hypothesis that M_{LogOR}=1 (meaning that there is no association between anxiety and cannabis use/CUD) according to the following formula:

$$Z = \frac{M_{LogOR}}{SE_{MLogOR}}$$

In the final step of the analysis the overall mean weighted effect size (M_{LogOR}) and its *95%CI* were antiloged as follows [1, p. 97]:

$$M_{LogOR} = e^{MLogOR}$$

$$\text{Lower } (95\%CI_{MLogOR}) = e^{LogLowerMLogOR}$$

$$\text{Upper } (95\%CI_{MLogOR}) = e^{LogUpperMLogOR}$$

Publication bias analyses in Comprehensive Meta-Analysis (CMA) software

Publication bias refers to an overestimation of the overall mean weighted effect size in meta-analysis due to inclusion of studies based on large sample sizes and/or large effect sizes [1, Chapter 30]. Such studies are more likely to be published and thus are easier to locate during a systematic search than studies based on smaller samples and/or small (often not statistically significant) effect sizes that are either not published at all or published in smaller (often non-English language) journals that are not included in major databases [1, Chapter 30].

Publication bias in the current study was assessed using methods available in the Comprehensive Meta-Analysis (CMA) software, version 2.2 (Biostat Inc., Englewood, NJ, USA). The theoretical number of null-studies (with *OR*=1) required to remove the statistical significance of the overall mean weighted *OR* in meta-analysis was computed using Rosenthal's Fail-Safe *N* [5]. The smaller the Fail-Safe *N*, the more likely it is that publication bias is present in meta-analysis.

Publication bias can also be assessed visually using a funnel plot of *LogOR* vs. *SEM* [6]. According to the funnel plot, the distribution of all effect sizes around the overall mean weighted *LogOR* should resemble a symmetrical funnel. Since the Y-axis is reversed (smaller *SEM* values on top, larger on the bottom of the plot), it was expected that larger studies with smaller variability would be found towards the top of the plot, close to and on both sides of the overall mean weighted *LogOR*. The small studies with larger variability would be found towards the bottom of the plot and they would spread wider away from and on both sides of the overall mean weighted *LogOR.* Such symmetrical funnel plot would indicate that some studies in the current meta-analysis show that anxiety and cannabis use/CUD are positively associated while others show either no association or a negative association. Any deviation

from such symmetry towards the right or the left of the overall mean weighted *LogOR* would indicate presence of publication bias in the current analysis.

Because a visual inspection of the funnel plot is subjective, the Duval and Tweedie's Trim-and-Fill analysis [7] was used to test for symmetry in such plot using mathematical assumptions of symmetry. Specifically, first the extreme studies from one side of the plot are removed ('trimmed') until the plot becomes symmetrical. This procedure adjusts the overall mean weighted *LogOR.* Then, the studies are added ('filled') back onto the plot and a mirror image of each one is produced and added to the opposite side of the plot to maintain symmetry. This procedure corrects the variance of the new estimate of the overall mean weighted *LogOR.* Publication bias is present if mostly the smaller studies towards the bottom of the plot are missing from the analysis and the adjusted overall mean weighted effect size differs from the original overall mean weighted effect size (for example, the effect size changes direction and/or its *95%CI* overlaps with the line of no effect following the adjustment for missing studies).

Finally, the results of two more methods were inspected in the current analysis. However, both methods are unreliable because they are based on the standard null-hypothesis testing and have low power (and thus high Type II error) if the number of studies in the analysis is low. Specifically, the Begg and Mazumdar Rank Order Correlation (Kendall's *tau b*) was used to investigate the relationship between the standardised effect sizes vs. *SEM* in each study [8] and the Egger's regression [9] was used to predict the standardised effect size with 1/*SEM*. Publication bias is present if smaller studies differ systematically (significantly) from the larger studies. In this case, either the correlation is statistically significant and/or the intercept of the regression line significantly deviates from zero causing the asymmetry of the funnel plot [9].

Table S1. Exclusion criteria applied to *N*=267 studies

Titles and abstracts of *N*=267 studies assessed for relevance (by LTL and KKK); *N*=218 excluded

Exclusion criteria:

- *N*=164 Irrelevant title/abstract
- *N*=28 Review/comment/no original data
- *N*=2 Healthy controls missing
- *N*=22 Non-users missing
- *N*=2 Unpublished thesis

Note. From the *N*=267 studies on the association between cannabis use and anxiety disorders, *N*=256 were located based on the electronic searches and *N*=11 from the hand search. A complete list of *N*=218 excluded studies and the individual reasons of exclusion are available upon request from the authors.

Table S2. Studies assessed in full length (*N*=49) and reasons for exclusion

Citation	Search type	Included (+) Excluded (–)	Reason for exclusion or inclusion comments
[10]	Hand search	+	
[11]	Hand search	+	in Moore *et al.* 2007 [12]
[13]	Search 1-2	–	Inadequate data (*SD* values missing)
[14]	Search 1-2	–	No anxiety diagnosis (anxiety sensitivity, anxious arousal)
[15]	Hand search	+	
[16]	Search 1-2	+	in Moore *et al.* 2007 [12]
[17]	Search 1-2	–	Non-users missing
[18]	Search 1-2	–	Healthy non-users missing
[19]	Search 1-2	+	
[20]	Search 1-2	+	
[21]	Search 1-2	–	Cannabis vs. anxiety comparison not shown
[22]	Search 1-2	+	
[23]	Search 1-2	+	
[24]	Search 1-2	+	
[25]	Search 1-2	+	
[26]	Search 1-2	–	Same cases as in [25]
[27]	Search 1-2	+	Anxiety and depression
[28]	Search 1-2	–	Cannabis vs. anxiety comparison not shown
[29]	Search 1-2	+	
[30]	Hand search	+	in Moore *et al.* 2007 [12]
[31]	Search 1-2	–	Cannabis vs. anxiety comparison not shown
[32]	Search 1-2	+	
[33]	Search 1-2	–	Same cases as in [34]
[34]	Search 1-2	+	
[35]	Hand search	+	Anxiety and depression
[36]	Search 1-2	–	High comorbidity with other substances (seekers of treatment for cannabis withdrawal)
[37]	Hand search	+	in Moore *et al.* 2007 [12]
[38]	Search 1-2	–	Cannabis vs. anxiety comparison not shown
[39]	Search 1-2	+	Anxiety and depression
[40]	Search 1-2	+	
[41]	Search 1-2	+	
[42]	Search 1-2	–	No anxiety diagnosis (anxiety sensitivity)
[43]	Hand search	+	
[44]	Hand search	+	Anxiety and depression
[45]	Search 1-2	–	Cannabis vs. anxiety comparison not shown
[46]	Search 1-2	–	Cannabis vs. anxiety comparison not shown
[47]	Search 1-2	+	Anxiety and depression (in Moore *et al.* 2007 [12])
[48]	Search 1-2	–	High comorbidity with other substances (music festivals attendees)
[49]	Search 1-2	+	
[50]	Hand search	+	Anxiety and depression
[51]	Hand search	+	
[52]	Search 1-2	+	

[53]	Search 1-2	–	Cases in treatment for cannabis dependence
[54]	Search 1-2	–	Inadequate data (too few anxiety cases to compute *OR*)
[55]	Search 1-2	–	Inadequate data (*SD* values missing)
[56]	Hand search	+	NPMS study methods; unpublished results in Moore *et al.* 2007 [12]
[57]	Search 1-2	+	
[58]	Search 1-2	+	
[59]	Search 1-2	+	

Note: The *N*=49 studies included *N*=38 from the electronic searches and *N*=11 from the hand search. A total of *N*=31 studies were selected for the final meta-analysis. All studies in the table above were inspected in full-length and assessed by both authors.
Abbreviations: NPMS: the British National Psychiatric Morbidity Survey, UK; *OR*: odds ratio; *SD*: standard deviation

Figure S1. Random-effects one-study removed analysis

a) Anxiety vs. cannabis use (*N*=15)

Study name	Statistics with study removed				
	Point	Lower limit	Upper limit	Z-Value	p-Value
Crum et al. 1993 [30]	1.23	1.05	1.45	2.53	0.011
Fergusson et al. 1996 [37]	1.24	1.06	1.46	2.66	0.008
Brook et al. 1998 [15]	1.26	1.04	1.51	2.41	0.016
Brook et al. 2001 [16] combined	1.25	1.05	1.50	2.47	0.014
Degenhardt et al. 2001 [34]	1.28	1.09	1.50	3.03	0.002
Chabrol et al. 2005 [25]	1.24	1.05	1.46	2.51	0.012
Lamers et al. 2006 [40]	1.26	1.07	1.47	2.87	0.004
Zvolensky et al. 2006 [58]	1.28	1.09	1.50	3.04	0.002
van Laar et al. 2007 [52]	1.25	1.06	1.47	2.62	0.009
Wittchen et al. 2007 [57]	1.22	1.03	1.44	2.33	0.020
Buckner & Schmidt 2008 [20]	1.28	1.09	1.50	3.04	0.002
Chabrol et al. 2008 [24]	1.21	1.04	1.42	2.40	0.016
NCS-R 2010 [29, 49] combined	1.17	1.03	1.34	2.38	0.017
Buckner et al. 2012 [19]	1.26	1.07	1.48	2.72	0.007
Degenhardt et al. 2013 [32]	1.22	1.05	1.42	2.59	0.010
	1.24	1.06	1.45	2.74	0.006

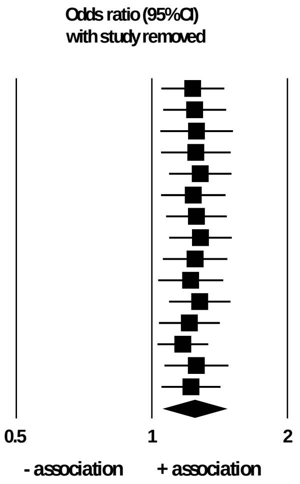

b) Anxiety vs. cannabis use disorder (CUD; *N*=13)

Study name	Statistics with study removed				
	Point	Lower limit	Upper limit	Z-Value	p-Value
Degenhardt et al. 2001 [34]	1.72	1.22	2.43	3.07	0.002
Agosti et al. 2002 [10]	1.61	1.17	2.21	2.95	0.003
Chabrol et al. 2005 [25]	1.69	1.21	2.37	3.07	0.002
Beard et al. 2006 [11]	1.73	1.25	2.39	3.33	0.001
Zvolensky et al. 2006 [58]	1.65	1.19	2.29	3.00	0.003
Roberts et al. 2007 [49]	1.76	1.26	2.46	3.34	0.001
Wittchen et al. 2007 [57]	1.68	1.19	2.38	2.95	0.003
Buckner et al. 2008 [22]	1.61	1.18	2.19	3.00	0.003
Low et al. 2008 [41]	1.70	1.23	2.35	3.19	0.001
Cascone et al. 2011 [23]	1.85	1.45	2.36	4.97	0.000
Martins & Gorelick 2011 [43]	1.56	1.17	2.08	3.03	0.002
Degenhardt et al. 2013 [32]	1.65	1.19	2.28	2.99	0.003
van der Pol et al. 2013 [51]	1.73	1.24	2.42	3.24	0.001
	1.68	1.23	2.31	3.25	0.001

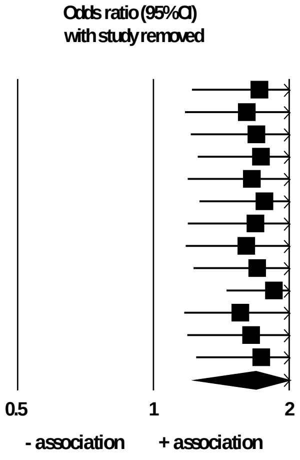

c) Anxiety+depression vs. cannabis use (N=5)

Study name	Statistics with study removed				
	Point	Lower limit	Upper limit	Z-Value	p-Value
McGee et al. 2000 [44]	1.52	1.02	2.28	2.04	0.042
Hayatbakhsh et al. 2007 [39]	1.59	1.03	2.45	2.09	0.037
NPMS 2007 [12]	1.92	1.47	2.51	4.83	0.000
Cheung et al. 2010 [27]	1.56	0.99	2.49	1.90	0.058
VAHCS 2010 [35, 47, 50] combined	1.77	1.14	2.75	2.55	0.011
	1.68	1.17	2.40	2.84	0.004

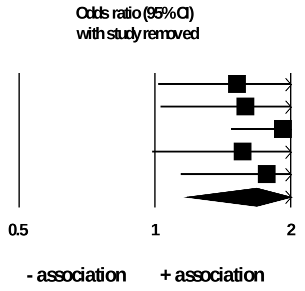

Note. 'Point' refers to the overall mean weighted effect size (*OR*) of all studies without the study in each row.

Figure S2. Random-effects cumulative analysis

a) Anxiety vs. cannabis use (*N*=15)

Study name	Point	Lower limit	Upper limit	Z-Value	p-Value
	Cumulative statistics				
Crum et al. 1993 [30]	1.54	0.78	3.04	1.23	0.218
Fergusson et al. 1996 [37]	1.40	0.82	2.39	1.22	0.221
Brook et al. 1998 [15]	1.18	1.02	1.36	2.20	0.028
Brook et al. 2001 [16] combined	1.18	1.04	1.33	2.62	0.009
Degenhardt et al. 2001 [34]	1.15	1.02	1.29	2.30	0.022
Chabrol et al. 2005 [25]	1.16	1.03	1.29	2.52	0.012
Lamers et al. 2006 [40]	1.15	1.03	1.29	2.43	0.015
Zvolensky et al. 2006 [58]	1.12	1.01	1.25	2.13	0.033
van Laar et al. 2007 [52]	1.13	1.01	1.25	2.22	0.026
Wittchen et al. 2007 [57]	1.16	1.05	1.28	2.87	0.004
Buckner & Schmidt 2008 [20]	1.14	1.02	1.27	2.34	0.019
Chabrol et al. 2008 [24]	1.16	1.02	1.32	2.30	0.022
NCS-R 2010 [29, 49] combined	1.23	1.05	1.44	2.55	0.011
Buckner et al. 2012 [19]	1.22	1.05	1.42	2.59	0.010
Degenhardt et al. 2013 [32]	1.24	1.06	1.45	2.74	0.006
	1.24	1.06	1.45	2.74	0.006

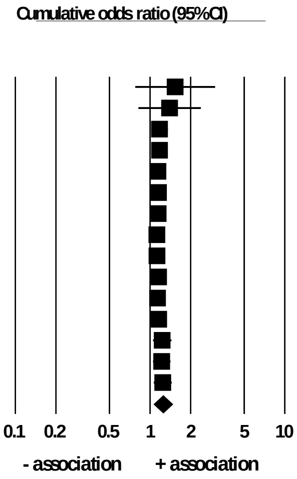

b) Anxiety vs. cannabis use disorder (CUD; *N*=13)

Study name	Point	Lower limit	Upper limit	Z-Value	p-Value
	Cumulative statistics				
Degenhardt et al. 2001 [34]	1.40	0.83	2.35	1.27	0.204
Agosti et al. 2002 [10]	1.89	1.03	3.48	2.07	0.039
Chabrol et al. 2005 [25]	1.80	1.23	2.65	3.01	0.003
Beard et al. 2006 [11]	1.71	1.17	2.50	2.75	0.006
Zvolensky et al. 2006 [58]	1.79	1.33	2.41	3.82	0.000
Roberts et al. 2007 [49]	1.63	1.18	2.25	2.99	0.003
Wittchen et al. 2007 [57]	1.67	1.31	2.12	4.14	0.000
Buckner et al. 2008 [22]	1.73	1.32	2.28	3.95	0.000
Low et al. 2008 [41]	1.72	1.34	2.21	4.25	0.000
Cascone et al. 2011 [23]	1.55	1.13	2.12	2.71	0.007
Martins & Gorelick 2011 [43]	1.70	1.20	2.40	2.98	0.003
Degenhardt et al. 2013 [32]	1.73	1.24	2.42	3.24	0.001
van der Pol et al. 2013 [51]	1.68	1.23	2.31	3.25	0.001
	1.68	1.23	2.31	3.25	0.001

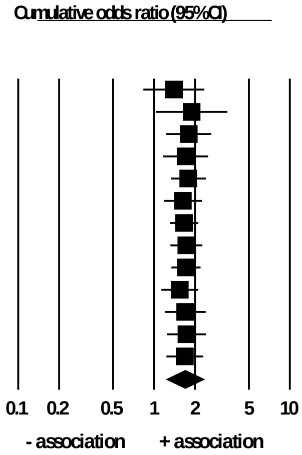

c) Anxiety+depression vs. cannabis use (*N*=5)

Study name	Cumulative statistics				
	Point	Lower limit	Upper limit	Z-Value	p-Value
McGee et al. 2000 [44]	2.45	1.41	4.25	3.17	0.002
Hayatbakhsh et al. 2007 [39]	2.29	1.51	3.49	3.87	0.000
NPMS 2007 [12]	1.64	0.85	3.17	1.47	0.142
Cheung et al. 2010 [27]	1.77	1.14	2.75	2.55	0.011
VAHCS 2010 [35, 47, 50] combined	1.68	1.17	2.40	2.84	0.004
	1.68	1.17	2.40	2.84	0.004

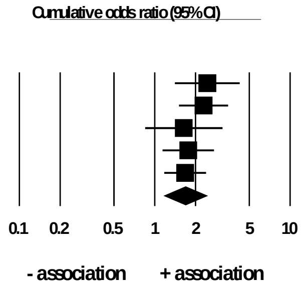

Note. 'Point' refers to the overall mean weighted effect size (*OR*) of all studies up to and including a study in that row.

Figure S3. Random-effects subgroup analyses

a) Anxiety vs. cannabis use (*N*=15)- comparing studies with *OR* adjusted for substance use/other illnesses/demographics vs. studies with unadjusted *OR*

Group by OR adjusted	Study name	Odds ratio	Lower limit	Upper limit	Z-Value	p-Value
no	Chabrol et al. 2005 [25]	1.36	0.83	2.22	1.22	0.222
no	Buckner & Schmidt 2008 [20]	0.81	0.50	1.33	-0.83	0.409
no	Chabrol et al. 2008 [24]	1.94	1.14	3.30	2.43	0.015
no		1.28	0.78	2.08	0.98	0.326
yes	Crum et al. 1993 [30]	1.54	0.78	3.04	1.23	0.218
yes	Fergusson et al. 1996 [37]	1.20	0.51	2.84	0.41	0.678
yes	Brook et al. 1998 [15]	1.16	1.00	1.35	1.94	0.053
yes	Brook et al. 2001 [16] combined	1.18	0.94	1.48	1.43	0.153
yes	Degenhardt et al. 2001 [34]	0.88	0.60	1.29	-0.65	0.513
yes	Lamers et al. 2006 [40]	0.55	0.15	2.03	-0.90	0.368
yes	Zvolensky et al. 2006 [58]	0.89	0.62	1.28	-0.63	0.528
yes	van Laar et al. 2007 [52]	1.18	0.71	1.97	0.64	0.525
yes	Wittchen et al. 2007 [57]	1.50	1.09	2.07	2.46	0.014
yes	NCS-R 2010 [29, 49] combined	2.04	1.50	2.78	4.53	0.000
yes	Buckner et al. 2012 [19]	1.05	0.62	1.80	0.19	0.853
yes	Degenhardt et al. 2013 [32]	3.20	1.11	9.25	2.15	0.032
yes		1.24	1.04	1.47	2.46	0.014
Overall		1.24	1.06	1.46	2.64	0.008

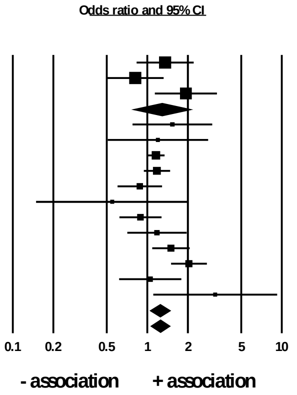

b) Anxiety vs. cannabis use (*N*=15)- comparing studies with vs. without clinical diagnoses of anxiety

Group by Clinical diagnosis	Studyname	Odds ratio	Lower limit	Upper limit	Z-Value	p-Value
no	Brook et al. 2001 [16] combined	1.18	0.94	1.48	1.43	0.153
no	Chabrol et al. 2005 [25]	1.36	0.83	2.22	1.22	0.222
no	Lamers et al. 2006 [40]	0.55	0.15	2.03	-0.90	0.368
no	Buckner & Schmidt 2008 [20]	0.81	0.50	1.33	-0.83	0.409
no	Chabrol et al. 2008 [24]	1.94	1.14	3.30	2.43	0.015
no	Buckner et al. 2012 [19]	1.05	0.62	1.80	0.19	0.853
no		1.17	0.93	1.48	1.32	0.186
yes	Crum et al. 1993 [30]	1.54	0.78	3.04	1.23	0.218
yes	Fergusson et al. 1996 [37]	1.20	0.51	2.84	0.41	0.678
yes	Brook et al. 1998 [15]	1.16	1.00	1.35	1.94	0.053
yes	Degenhardt et al. 2001 [34]	0.88	0.60	1.29	-0.65	0.513
yes	Zvolensky et al. 2006 [58]	0.89	0.62	1.28	-0.63	0.528
yes	van Laar et al. 2007 [52]	1.18	0.71	1.97	0.64	0.525
yes	Wittchen et al. 2007 [57]	1.50	1.09	2.07	2.46	0.014
yes	NCS-R 2010 [29, 49] combined	2.04	1.50	2.78	4.53	0.000
yes	Degenhardt et al. 2013 [32]	3.20	1.11	9.25	2.15	0.032
yes		1.29	1.04	1.61	2.30	0.021
Overall		1.24	1.05	1.45	2.58	0.010

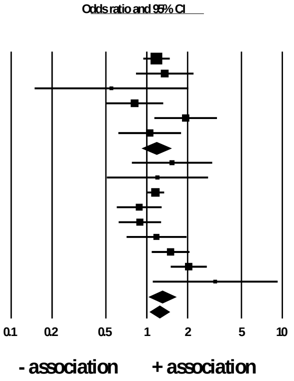

c) Anxiety vs. cannabis use disorder (CUD; *N*=13)- comparing studies with *OR* adjusted for substance use/other illnesses/demographics vs. studies with unadjusted *OR*

Group by OR adjusted	Study name	Odds ratio	Lower limit	Upper limit	Z-Value	p-Value
no	Agosti et al. 2002 [10]	2.60	1.50	4.50	3.41	0.001
no	Chabrol et al. 2005 [25]	1.61	0.84	3.07	1.43	0.151
no	Beard et al. 2006 [11]	0.78	0.18	3.30	-0.34	0.735
no		1.86	1.10	3.15	2.31	0.021
yes	Degenhardt et al. 2001 [34]	1.40	0.83	2.35	1.27	0.204
yes	Zvolensky et al. 2006 [58]	2.10	1.06	4.15	2.13	0.033
yes	Roberts et al. 2007 [49]	0.90	0.39	2.06	-0.25	0.803
yes	Wittchen et al. 2007 [57]	1.70	1.13	2.56	2.53	0.011
yes	Buckner et al. 2008 [22]	4.88	1.43	16.65	2.53	0.011
yes	Low et al. 2008 [41]	1.40	0.41	4.80	0.54	0.592
yes	Cascone et al. 2011 [23]	1.02	0.97	1.08	0.72	0.470
yes	Martins & Gorelick 2011 [43]	3.20	1.98	5.16	4.77	0.000
yes	Degenhardt et al. 2013 [32]	2.20	1.10	4.40	2.23	0.026
yes	van der Pol et al. 2013 [51]	1.12	0.48	2.62	0.26	0.794
yes		1.67	1.17	2.37	2.83	0.005
Overall		1.72	1.29	2.31	3.64	0.000

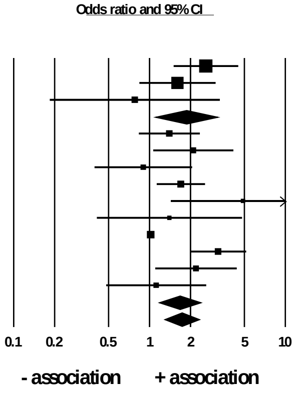

d) Anxiety vs. cannabis use disorder (CUD; *N*=13)- clinical diagnoses

Group by Clinical diagnosis	Study name	Odds ratio	Lower limit	Upper limit	Z-Value	p-Value
no	Chabrol et al. 2005 [25]	1.61	0.84	3.07	1.43	0.151
no	Cascone et al. 2011 [23]	1.02	0.97	1.08	0.72	0.470
no		1.14	0.78	1.66	0.66	0.509
yes	Degenhardt et al. 2001 [34]	1.40	0.83	2.35	1.27	0.204
yes	Agosti et al. 2002 [10]	2.60	1.50	4.50	3.41	0.001
yes	Beard et al. 2006 [11]	0.78	0.18	3.30	-0.34	0.735
yes	Zvolensky et al. 2006 [58]	2.10	1.06	4.15	2.13	0.033
yes	Roberts et al. 2007 [49]	0.90	0.39	2.06	-0.25	0.803
yes	Wittchen et al. 2007 [57]	1.70	1.13	2.56	2.53	0.011
yes	Buckner et al. 2008 [22]	4.88	1.43	16.65	2.53	0.011
yes	Low et al. 2008 [41]	1.40	0.41	4.80	0.54	0.592
yes	Martins & Gorelick 2011 [43]	3.20	1.98	5.16	4.77	0.000
yes	Degenhardt et al. 2013 [32]	2.20	1.10	4.40	2.23	0.026
yes	van der Pol et al. 2013 [51]	1.12	0.48	2.62	0.26	0.794
yes		1.87	1.43	2.44	4.60	0.000
Overall		1.58	1.27	1.97	4.14	0.000

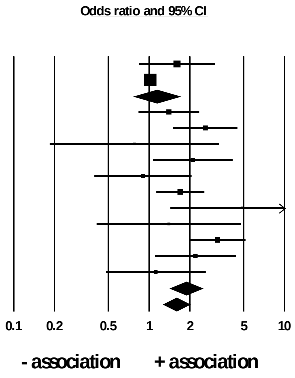

Bibliography

1. Borenstein M, Hedges L, Higgins J, Rothstein H: ***Introduction to meta-analysis***. Great Britain: John Wiley & Sons, Ltd; 2009.
2. Bland J, Altman D: **Statistics Notes: The odds ratio.** *Br Med J* 2000, **320:**1468.
3. ***Comprehensive Meta Analysis (CMA 2.2, Help Manual)***. Englewood, NJ, USA: Biostat Inc; 2011.
4. DerSimonian R, Laird N: **Meta-analysis in clinical trials.** *Control Clin Trials* 1986, **7:**177-188.
5. Rosenthal R: **The 'file drawer' problem and tolerance for null results.** *Psychol Bull* 1979, **86:**638-641.
6. Sterne J, Egger M: **Funnel plots for detecting bias in meta-analysis: guidelines on choice of axis.** *J Clin Epidemiol* 2001, **54:**1046-1055.
7. Duval S, Tweedie R: **A nonparametric 'trim and fill' method of accounting for publication bias in meta-analysis.** *J Am Statist Assoc* 2000, **95:**89-98.
8. Begg C, Mazumdar M: **Operating characterstics of a rank correlation test for publication bias** *Biometrics* 1994, **50:**1088-1101.
9. Egger M, Davey S, Schneider M, Minder C: **Bias in meta-analysis detected by a simple graphical test.** *Br Med J* 1997, **315:**629-634.
10. Agosti V, Nunes E, Levin F: **Rates of psychiatric comorbidity among U.S. residents with lifetime cannabis dependence.** *Am J Drug Alcohol Abuse* 2002, **28:**643-652.
11. Beard JR, Dietrich UC, Brooks LO, Brooks RT, Heathcote K, Kelly B: **Incidence and outcomes of mental disorders in a regional population: the Northern Rivers Mental Health Study.** *Aust N Z J Psychiatry* 2006, **40:**674-682.
12. Moore THM, Zammit S, Lingford-Hughes A, Barnes TRE, Jones PB, Burke M, Lewis G: **Cannabis use and risk of psychotic or affective mental heath outcomes: A systematic review.** *Lancet* 2007, **370:**319-328.
13. Bonn-Miller MO, Zvolensky MJ, Johnson KA: **Uni-morbid and co-occurring marijuana and tobacco use: Examination of concurrent associations with negative mood states.** *J Addict Dis* 2010, **29:**68-77.
14. Bonn-Miller MO, Zvolensky MJ, Leen-Feldner EW, Feldner MT, Yartz AR: **Marijuana use among daily tobacco smokers: Relationship to anxiety-related factors.** *J Psychopathol Behav Assess* 2005, **27:**279-289.
15. Brook J, Cohen P, Brook D: **Longitudinal study of co-occurring psychiatric disorders and substance use.** *J Am Acad Child Adolesc Psychiatry* 1998, **37:**322-330.

16. Brook JS, Rosen Z, Brook DW: **The effect of early marijuana use on later anxiety and depressive symptoms.** *NYS Psychologist* 2001, **13:**35-40.
17. Buckner JD, Bonn-Miller MO, Zvolensky MJ, Schmidt NB: **Marijuana use motives and social anxiety among marijuana-using young adults.** *Addict Behav* 2007, **32:**2238-2252.
18. Buckner JD, Heimberg RG, Schneier FR, Liu S-M, Wang S, Blanco C: **The relationship between cannabis use disorders and social anxiety disorder in the National Epidemiological Study of Alcohol and Related Conditions (NESARC).** *Drug Alcohol Depend* 2012, **124:**128-134.
19. Buckner JD, Joiner TE, Jr., Schmidt NB, Zvolensky MJ: **Daily marijuana use and suicidality: The unique impact of social anxiety.** *Addict Behav* 2012, **37:**387-392.
20. Buckner JD, Schmidt NB: **Marijuana effect expectancies: Relations to social anxiety and marijuana use problems.** *Addict Behav* 2008, **33:**1477-1483.
21. Buckner JD, Schmidt NB, Bobadilla L, Taylor J: **Social anxiety and problematic cannabis use: Evaluating the moderating role of stress reactivity and perceived coping.** *Behav Res Ther* 2006, **44:**1007-1015.
22. Buckner JD, Schmidt NB, Lang AR, Small JW, Schlauch RC, Lewinsohn PM: **Specificity of social anxiety disorder as a risk factor for alcohol and cannabis dependence.** *J Psychiatr Res* 2008, **42:**230-239.
23. Cascone P, Zimmermann G, Auckenthaler B, Robert-Tissot C: **Cannabis dependence in Swiss adolescents: Exploration of the role of anxiety, coping styles, and psychosocial difficulties.** *Swiss J Psychol* 2011, **70:**129-139.
24. Chabrol H, Chauchard E, Girabet J: **Cannabis use and suicidal behaviours in high-school students.** *Addict Behav* 2008, **33:**152-155.
25. Chabrol H, Ducongé E, Casas C, Roura C, Carey KB: **Relations between cannabis use and dependence, motives for cannabis use and anxious, depressive and borderline symptomatology.** *Addict Behav* 2005, **30:**829-840.
26. Chabrol H, Duconge E, Roura C, Casas C: **[Relations between anxious, depressive and borderline symptomatology and frequency of cannabis use and dependence].** *L'encéphale* 2004, **30:**141-146.
27. Cheung JTW, Mann RE, Ialomiteanu A, Stoduto G, Chan V, Ala-Leppilampi K, Rehm J: **Anxiety and mood disorders and cannabis use.** *Am J Drug Alcohol Abuse* 2010, **36:**118-122.

28. Cohen P, Cohen J, Kasen S, Velez CN: **An epidemiological study of disorders in late childhood and adolescence: I. Age- and gender-specific prevalence.** *J Child Psychol Psychiatry* 1993, **34:**851-867.
29. Cougle JR, Bonn-Miller MO, Vujanovic AA, Zvolensky MJ, Hawkins KA: **Posttraumatic stress disorder and cannabis use in a nationally representative sample.** *Psychol Addict Behav* 2011, **25:**554-558.
30. Crum RM, Anthony JC: **Cocaine use and other suspected risk factors for obsessive-compulsive disorder: a prospective study with data from the Epidemiologic Catchment Area surveys.** *Drug Alcohol Depend* 1993, **31:**281-295.
31. de Dios MA, Hagerty CE, Herman DS, Hayaki J, Anderson BJ, Budney AJ, Stein M: **General anxiety disorder symptoms, tension reduction, and marijuana use among young adult females.** *J Womens Health* 2010, **19:**1635-1642.
32. Degenhardt L, Coffey C, Romaniuk H, Swift W, Carlin JB, Hall WD, Patton GC: **The persistence of the association between adolescent cannabis use and common mental disorders into young adulthood.** *Addiction* 2013, **108:**124-133.
33. Degenhardt L, Hall W, Lynskey M: **Alcohol, cannabis and tobacco use among Australians: A comparison of their associations with other drug use and use disorders, affective and anxiety disorders, and psychosis.** *Addiction* 2001, **96:**1603-1614.
34. Degenhardt L, Hall W, Lynskey M: **The relationship between cannabis use, depression and anxiety among Australian adults: Findings from the National Survey of Mental Health and Well-Being.** *Soc Psychiatry Psychiatr Epidemiol* 2001, **36:**219-227.
35. Degenhardt L, Coffey C, Carlin JB, Swift W, Moore E, Patton GC: **Outcomes of occasional cannabis use in adolescence: 10-year follow-up study in Victoria, Australia.** *Br J Psychiatry* 2010, **196:**290-295.
36. Dorard G, Berthoz S, Phan O, Corcos M, Bungener C: **Affect dysregulation in cannabis abusers: a study in adolescents and young adults.** *Eur Child Adolesc Psychiatry* 2008, **17:**274-282.
37. Fergusson DM, Lynskey MT, Horwood LJ: **The short-term consequences of early onset cannabis use.** *J Abnorm Child Psychol* 1996, **24:**499-512.
38. Gilder DA, Lau P, Dixon M, Corey L, Phillips E, Ehlers CL: **Co-morbidity of select anxiety, affective, and psychotic disorders with cannabis dependence in Southwest California Indians.** *J Addict Dis* 2006, **25:**67-79.

39. Hayatbakhsh MR, Najman JM, Jamrozik K, Mamun AA, Alati R, Bor W: **Cannabis and anxiety and depression in young adults: A large prospective study.** *J Am Acad Child Adolesc Psychiatry* 2007, **46:**408-417.
40. Lamers CTJ, Bechara A, Rizzo M, Ramaekers JG: **Cognitive function and mood in MDMA/THC users, THC users and non-drug using controls.** *J Psychopharmacol (Oxf)* 2006, **20:**302-311.
41. Low NC, Lee SS, Johnson JG, Williams JB, Harris ES: **The association between anxiety and alcohol versus cannabis abuse disorders among adolescents in primary care settings.** *Fam Pract* 2008, **25:**321-327.
42. Malmberg M, Overbeek G, Monshouwer K, Lammers J, Vollebergh WAM, Engels RCME: **Substance use risk profiles and associations with early substance use in adolescence.** *J Behav Med* 2010, **33:**474-485.
43. Martins S, Gorelick D: **Conditional substance abuse and dependence by diagnosis of mood or anxiety disorder or schizophrenia in the U.S. population.** *Drug Alcohol Depend* 2011, **119:**28-36.
44. McGee R, Williams S, Poulton R, Moffitt T: **A longitudinal study of cannabis use and mental health from adolescence to early adulthood.** *Addiction* 2000, **95:**491-503.
45. Monshouwer K, Van Dorsselaer S, Verdurmen J, Bogt TT, De Graaf R, Vollebergh W: **Cannabis use and mental health in secondary school children. Findings from a Dutch survey.** *Br J Psychiatry* 2006, **188:**148-153.
46. Newbury-Birch D, Lowry RJ, Kamali F: **The changing patterns of drinking, illicit drug use, stress, anxiety and depression in dental students in a UK dental school: a longitudinal study.** *Br Dent J* 2002, **192:**646-649.
47. Patton GC, Coffey C, Carlin JB, Degenhardt L, Lynskey M, Hall W: **Cannabis use and mental health in young people: cohort study.** *Br Med J* 2002, **325:**1195-1198.
48. Pavarin RM: **Substance use and related problems: a study on the abuse of recreational and not recreational drugs in Northern Italy.** *Ann Ist Super Sanita* 2006, **42:**477-484.
49. Roberts RE, Roberts CR, Xing Y: **Rates of DSM-IV psychiatric disorders among adolescents in a large metropolitan area.** *J Psychiatr Res* 2007, **41:**959-967.
50. Swift W, Coffey C, Carlin JB, Degenhardt L, Patton GC: **Adolescent cannabis users at 24 years: trajectories to regular weekly use and dependence in young adulthood.** *Addiction* 2008, **103:**1361-1370.

51. van der Pol P, Liebregts N, de Graaf R, Ten Have M, Korf D, van den Brink W, van Laar M: **Mental health differences between frequent cannabis users with and without dependence and the general population.** *Addiction* 2013, **DOI: 10.1111/add.12196**.

52. van Laar M, van Dorsselaer S, Monshouwer K, de Graaf R: **Does cannabis use predict the first incidence of mood and anxiety disorders in the adult population?** *Addiction* 2007, **102:**1251-1260.

53. Wagner MF, da Silva Oliveira M: **Estudo das habilidades sociais em adolescentes usuários de maconha.** *Psicologia em Estudo* 2009, **14:**101-110.

54. Weller RA, Halikas JA: **Marijuana use and psychiatric illness: a follow-up study.** *Am J Psychiatry* 1985, **142:**848-850.

55. Wells B, Stacey B: **A further comparison of cannabis (marijuana) users and non-users.** *Br J Addict* 1976, **71:**161-165.

56. Wiles N, Zammit S, Bebbington P, Singleton N, Meltzer H, Lewis G: **Self-reported psychotic symptoms in the general population. Results from the longitudinal study of the British National Psychiatric Morbidity Survey.** *Br J Psychiatry* 2006, **188:**519-526.

57. Wittchen H-U, Fröhlich C, Behrendt S, Günther A, Rehm J, Zimmermann P, Lieb R, Perkonigg A: **Cannabis use and cannabis use disorders and their relationship to mental disorders: A 10-year prospective-longitudinal community study in adolescents.** *Drug Alcohol Depend* 2007, **88:**S60-S70.

58. Zvolensky MJ, Bernstein A, Sachs-Ericsson N, Schmidt NB, Buckner JD, Bonn-Miller MO: **Lifetime associations between cannabis, use, abuse, and dependence and panic attacks in a representative sample.** *J Psychiatr Res* 2006, **40:**477-486.

59. Zvolensky MJ, Cougle JR, Johnson KA, Bonn-Miller MO, Bernstein A: **Marijuana use and panic psychopathology among a representative sample of adults.** *Exp Clin Psychopharmacol* 2010, **18:**129-134.
